# Small GTPase RAB6 deficiency promotes alveolar progenitor cell renewal and attenuates PM2.5-induced lung injury and fibrosis

**DOI:** 10.1038/s41419-020-03027-2

**Published:** 2020-10-04

**Authors:** Lawei Yang, Gang Liu, Xiaomin Li, Zhengyuan Xia, Yahong Wang, Weihao Lin, Wei Zhang, Wenjuan Zhang, Xuenong Li

**Affiliations:** 1 Department of Pathology, School of Basic Medical Sciences, Southern Medical University, 510515 Guangzhou, China; 2 Clinical Research Center, Affiliated Hospital of Guangdong Medical University, 524001 Zhanjiang, China; 3 Department of Anesthesiology, Affiliated Hospital of Guangdong Medical University, 524001 Zhanjiang, China; 4 Department of Anesthesiology, The University of Hong Kong, Hong Kong, China

**Keywords:** Self-renewal, Chronic inflammation

## Abstract

Idiopathic pulmonary fibrosis (IPF) is a progressive interstitial lung disease characterized by chronic non-specific inflammation of the interstitial lung and extensive deposition of collagen fibers leading to destruction of lung function. Studies have demonstrated that exposure to fine particulate matter (PM2.5) increases the risk of IPF. In order to recover from PM2.5-induced lung injury, alveolar epithelial cells need to be repaired and regenerated to maintain lung function. Type 2 alveolar epithelial cells (AEC2) are stem cells in the adult lung that contribute to the lung repair process through complex signaling. Our previous studies demonstrated that RAB6, a RAS family member lowly expressed in lung cancer, inhibited lung cancer stem cell self-renewal, but it is unclear whether or not and how RAB6 may regulate AEC2 cell proliferation and self-renewal in PM2.5-induced pulmonary fibrosis. Here, we demonstrated that knockout of RAB6 inhibited pulmonary fibrosis, oxidative stress, and AEC2 cell death in PM2.5-injured mice. In addition, knockout of RAB6 decreased Dickkopf 1(DKK1) autocrine and activated proliferation, self-renewal, and wnt/β-catenin signaling of PM2.5-injured AEC2 cells. RAB6 overexpression increased DKK1 autocrine and inhibited proliferation, self-renewal and wnt/β-catenin signaling in AEC2 cells in vitro. Furthermore, DKK1 inhibitors promoted proliferation, self-renewal and wnt/β-catenin signaling of RAB6 overexpressing AEC2 cells, and attenuated PM2.5-induced pulmonary fibrosis in mice. These data establish RAB6 as a regulator of DKK1 autocrine and wnt/β-catenin signal that serves to regulate AEC2 cell proliferation and self-renewal, and suggest a mechanism that RAB6 disruption may promote AEC2 cell proliferation and self-renewal to enhance lung repair following PM2.5 injury.

## Introduction

Idiopathic pulmonary fibrosis (IPF) is a progressive interstitial lung disease characterized by chronic non-specific inflammation of the interstitial lung and extensive deposition of collagen fibers leading to destruction of lung function^1^. Unlike other forms of lung disease, the pathogenesis of IPF is poorly understood. There is currently no effective treatment for this fatal disease, which has a higher mortality rate than most tumors and is called a “tumor-like disease”^2^. The incidence and mortality from IPF is increasing worldwide, with a median survival of only 3–4 years. The recently approved drugs for IPF, such as Nintedanib and Pirfenidone, can reduce the occurrence of lung dysfunction, but no drugs so far have been shown to improve survival or the quality of life^3,4^.

In recent years, the incidence of pulmonary fibrosis has increased with the increasing severity of air pollution. Epidemiological studies have shown that the increase in the incidence of pulmonary fibrosis is associated with increasing fine particulate matter (PM2.5, aerodynamic diameter ≤ 2.5 μm) levels in the atmosphere^5^. Due to its small diameter, PM2.5 can directly reach the alveoli or even the blood after being inhaled. It can damage antioxidant enzymes and proteins through the harmful components adsorbed, and it can also cause damage through increasing reactive oxygen species (ROS), which destroys the functions of the alveolar epithelial cells^6^. Our previous study showed that PM2.5 could cause DNA and mitochondrial damage and enhance autophagy in alveolar epithelial cells^7,8^. Further animal experiments showed that PM2.5 aggravates pulmonary fibrosis in mice through ROS/AKT signaling^9^. Recent studies show that long-term PM2.5 exposure can induce lung fibrosis in mice, which may be related to oxidative damage to PM2.5^10^. The specific molecular mechanism needs further research.

Alveolar stem cells, also known as alveolar progenitor cells, are cells that are capable of self-renewal and differentiate into functional lung tissue under specific conditions^11^. Type 2 alveolar epithelial cells (AEC2) are stem cells in the adult lung that contribute to the lung repair process^12^. It is now generally accepted that harmful stimulation of the alveoli can lead to loss of AEC2 or mutations in alveolar epithelial cells, thereby impairing their proliferation or self-renewal, which can trigger pulmonary fibrosis^13^. Recent studies have shown that promoting self-renewal of AEC2 cells alleviate the development of pulmonary fibrosis in mice^14^. Collectively, these observations point to AEC2s as central in the pathogenesis of pulmonary fibrosis, but the mechanisms whereby AEC2 cell renewal is regulated to modulate pulmonary fibrosis are incompletely understood.

Ras-related protein 6 (RAB6) is a kind of small GTPases which are involved in vesicular traffic in the secretory and endocytic pathways^15^. Rab protein, as a molecular switch of vesicle transport, interacts with its upstream regulatory factors and downstream specific effectors, plays an important role in the formation and transport of vesicles, and a series of Rab effector proteins have been found to be involved in cellular phagocytic and protein transport pathways^16–18^. RAB6 is involved in the composition of the Golgi and cytoplasmic vesicles and regulate protein transport between the Golgi apparatus, endoplasmic reticulum (ER) and endosomes^19,20^. Also, RAB6-mediated protein secretion is involved in the organization of cytoskeleton and the transport of mRNA^21,22^. In addition, RAB6 regulates the secretion of vesicles and affects the growth and development of zebrafish neurons^23^. Moreover, RAB6 protein is widely involved in various physiological activities such as cell adhesion and migration^24–27^. We have shown that RAB6 is down-regulated in lung cancer tissues, and its overexpression can inhibit cell proliferation and colony formation in non-small cell lung cancer^28^. Importantly, RAB6 is associated with self-renewal of lung cancer stem cells, and RAB6 can inhibit the expression of the cell stem genes *Sox2* and *Oct4*, and inhibit the clonal formation ability of lung stem cells^29^. In addition, we previously demonstrated that a large number of AEC2s underwent epithelial-mesenchymal transition and apoptosis in mice with pulmonary fibrosis and in IPF patients^30^. We, thus, hypothesized that RAB6 regulates the proliferation and self-renewal of AEC2s to modulate the development of pulmonary fibrosis.

Here, we show that RAB6 deficiency attenuates PM2.5-induced oxidative stress, alveolar epithelial cell death and lung fibrosis in mice. In addition, we found that knockout of RAB6 decreased Dickkopf 1(DKK1) autocrine and activated proliferation, self-renewal, and wnt/β-catenin signaling of PM2.5-injured AEC2 cells. Furthermore, we observed that DKK1 inhibition promotes proliferation, self-renewal and wnt/β-catenin signaling of RAB6 overexpressing AEC2 cells, and attenuates PM2.5-induced pulmonary fibrosis in mice.

## Materials and methods

### Animal experiment

C57BL/6J mice with knockout of RAB6 (RAB6^−/−^) have been described^31^. Six to eight-week old mice of equal male and female numbers were selected for study. All mice were bred and maintained in a pathogen-free environment with free access to food and water. All experiments were approved by the Ethics Committee of the Animal Experimental Center of Guangdong Medical University (No. GDY1801034).

For PM2.5 injury, 6–8-week old mice (WT or RAB6^−/−^) were randomly divided into two groups (*n* = 24), namely (WT or RAB6^−/−^) saline group and (WT or RAB6^−/−^) PM2.5 group. The mice in PM2.5 group were intratracheally instilled with 50 μl PM2.5 (100 mg/kg body weight) as described previously^9^. The collection, processing and composition testing of PM2.5 used in this study was as we reported previously^32^. The mice were instilled once per week for four weeks, and the lung injury and fibrosis-related test were performed 4 weeks after the last instillation. For DKK1 inhibitor treatment, mice were instilled with Gallocyanine (1 mg/kg body weight)(ab145230, Abcam) while instilling PM2.5 intratracheally.

### Human clinical samples

The study was approved by the Institutional Review Board of the Affiliated Hospital of Guangdong Medical University (No. PJ2012132), and was conducted under the guidance of its approval as we previously described^30^. Non-IPF lung tissues (Normal: paracancerous tissue of lung cancer) were obtained from the Department of Oncology, Affiliated Hospital of Guangdong Medical University. IPF lung tissues were obtained from lung transplant patients at the Affiliated Hospital of Nanjing University Medical School. Patients were told that their lung tissue was used for medical research and signed the “informed consent” of the project. The clinical data of the patients was described in Table 1.

**Table 1:** The clinical data of the patients.

Patient ID	Gender	Age	Smoking	DLCO(%)	FEV_1_(%)	FVC(%)
IPF1	Male	74	Yes	35.4	52.7	64.1
IPF2	Male	66	Yes	14.7	52.6	44.7
IPF3	Male	57	Yes	15.4	52.9	48.6
Non-IPF1	Male	71	Yes	/	/	/
Non-IPF2	Male	65	No	/	/	/
Non-IPF2	Female	54	Yes	/	/	/

*DLCO* diffusion capacity for carbon monoxide, *FEV*_*1*_ forced expiratory volume in 1 s, *FVC* forced vital capacity.

### Isolation, culture, and transfection of mouse AEC2 cells

Mouse AEC2 cells were enriched by surface marker sorting as previously reported^14^. Fresh mouse lung tissues were digested with Dispase and Collagenase at 37 °C for 20 min. Cells were resuspended and incubated with the antibody mixture anti-EPCAM(25–5791–80, eBioscience), anti-CD24(12–0242–82, eBioscience), anti-SFTPC(sc-518029, Santa Cruz), anti-CD31-CD34-CD45(13–0311–82, 13–0341–82, and 13-0451-82, eBioscience). The AEC2 cell population (CD24^−^ SFTPC^+^ subset) was isolated from the epithelial cell populations (EPCAM^+^CD31^−^CD34^−^CD35^−^) by the FACSAria sorter. The sorted AEC2 cells were seeded in a matrigel 6-well plate (354671, Corning, USA) and cultured in bronchial epithelial cell growth medium (BEGM) supplied with 1% FBS and growth factors (50 ng/mL FGF, 30 ng/mL HGF). The cell growth medium was changed every 2 days.

For PM2.5 injury, WT and RAB6^−/−^ AEC2 cells were exposed to PM2.5 (100 μg/ml) or saline for 48 h as we previously described^7^. For cell transfection, the RAB6 overexpression vector was constructed and transfected into AEC2 cells by Lipofectamine 2000 as we previously described^29^. For DDK1 protein treatment, WT and RAB6^−/−^ AEC2 cells were exposed to DKK1 protein (10 ng/ml) (ab205987, Abcam) or PBS for 48 h. For DKK1 inhibitor treatment, RAB6 overexpression (RAB6) and negative control (NC) AEC2 cells were exposed to DKK1 inhibitor (Gallocyanine, 5 μM) or PBS for 48 h.

### Immunofluorescence

Paraformaldehyde-fixed lung tissue or AEC2 cell samples were blocked and then incubated with primary antibodies RAB6 (9625, CST), SFTPC (sc-518029, Santa Cruz) or DKK1 (sc-374574, Santa Cruz) overnight. Next, the samples were incubated with FITC–labeled goat anti-rabbit antibody (31635, Invitrogen) and Alexa 647-conjugated goat anti-mouse antibody (A-21235, Invitrogen). Nuclear staining was performed with DAPI stain solution. Confocal images were captured using a Leica TCS SP8 confocal microscope.

### RNA isolation and quantitative real-time PCR (qRT-PCR)

Lung tissue or cells were lysed by TRIzol kit (QIAGEN) and RNA was isolated per the manufacturer’s instructions. In addition, the PCR was carried out by the One Step TB Green RT-PCR Kit (TaKaRa, Japan) as previously described. The relative expression of each gene was calculated using the 2^−ΔΔCT^ method after correction by GAPDH expression. All primer sequences are listed in the Supplemental Table 1.

### Histopathological analysis and immunohistochemistry

Lung tissues of all mice fixed in paraformaldehyde and embedded in paraffin were sectioned to a thickness of 5 μm. Then, the tissue slides were deparaffinized and rehydrated.

For lung collagen detection, the tissue slides were stained with MASSON trichrome stain kit as previously described^9^. After MASSON staining, the slides were dehydrated in gradient alcohol, sealed, and photographed under a light microscope.

For immunohistochemical detection of α-SMA or RAB6, the tissue slides were subjected to antigen retrieval, and the endogenous peroxidase was inactivated by treatment with H_2_O_2_ (3%). The slides were incubated with α-SMA (19245, CST) or RAB6 (9625, CST) antibodies, then washed and incubated with Biotin-labeled goat anti- rabbit IgG (65–6140, Invitrogen), followed by staining with the DAB substrate kit.

For TUNEL assay, the tissue slides were treated with proteinase K without DNase, and after washing, 50 μl of freshly prepared TUNEL assay solution (G3250, Promega) was added and incubated at 37 °C for 1 h in the dark. After nuclear staining, photographs were taken under a fluorescence microscope.

### Bronchoalveolar lavage fluid test

After the mice were sacrificed, the lungs were immediately lavaged with 0.9 mL of cold sterile PBS through a tracheal cannula to obtain BALF. The content of interleukin-1β (IL-1β) and tumor necrosis factor-α (TNF-α) in BALF was measured using sandwich enzyme-linked immunosorbent assays (ELISA) kit as we previously described^33^.

### Colony formation assays

AEC2 cells were seeded in a matrigel 6-well plate (354671, Corning, USA) and cultured for 2 weeks, and then the plate was fixed and stained with 0.1% crystal violet solution. Photographs were taken under the microscope and visible colonies were counted. The ability of cell clone formation was represented by the number of cell clones.

### Reactive oxygen species detection

The ROS assay was performed by DCFH-DA staining as we previously described^7^. Fresh mouse lung tissue was digested with Dispase and collagenase for 20 min at 37 °C to generate a cell suspension. The cells were suspended in the diluted DCFH-DA staining solution (10 μmol/L) (S0033S, Beyotime) and incubated at 37 °C for 20 min. Labeled cells were washed followed by flow cytometry detection.

### Transmission electron microscopy

The Transmission electron microscopy (TEM) assay was performed according to the TEM sample preparation protocol as we previously described^9^. Fixed lung tissue was embedded in eponate12, sliced and then double-stained with lead citrate and uranyl acetate. The samples were then photographed using a JEOL JEM-1400 transmission electron microscope.

### Co-Immunoprecipitation and western blotting

Protein A/G agarose beads were cross-linked with RAB6 antibody (9625, CST) or DKK1 antibody (sc-374574, Santa Cruz), respectively, according to the kit instructions. The cross-linked RAB6, DKK1 or IgG was added to each protein sample and incubated overnight at 4 °C followed by eluting in sample elution buffer. Finally, the protein that specifically binds to the cross-linked antibody complex was eluted for Western blotting.

For western blotting, proteins collected from Co-Immunoprecipitation (CO-IP) eluates, lung tissue or AEC2 cell lysates were separated by SDS-PAGE and PVDF membranes. Subsequently, blots were incubated with the primary antibodies (1:300) and then with secondary antibody (1:5000; 65–6140, Invitrogen). The blots were analyzed using a gel imaging system (BIO-RAD, USA). The bands grayscale values were quantified by ImageJ2X software. DKK1 (48367), Bax (5023), Bcl-2 (3498), Cleaved Caspase-3 (9664), wnt3a (2721), β-catenin (9582), c-Myc (5605), SOX2 (3579), Histone H3 (4499) and GAPDH (5174) were purchased from CST, 8-OHDG (sc-393871), PRDX5 (sc-133072), β-actin (sc-47778) were purchased from Santa Cruz, and OGG1 (NB100–106) was purchased from NOVUS (Supplemental Table 2).

### Statistical analysis

Data are shown as mean ± SEM. GraphPad Prism 6 (GraphPad Inc, USA) and SPSS 19.0 software (IBM Inc, USA) were used for statistical analysis of all data. Student’s *t* tests was used for the comparison between data from two groups. Statistically significant data are reported as indicated: *P < 0.05, **P < 0.01 and ***P < 0.001. Statistical analysis results and *P* values, as calculated through GraphPad Prism 6, are provided in Supplementary Table 3.

## Results

### RAB6 deficiency attenuated PM2.5-induced lung injury and fibrosis

We have recently shown that RAB6 inhibits the migration of lung cancer cells and inhibits the self-renewal and proliferation of lung cancer stem cells^28,29^. In this study, we sought to determine whether RAB6 could provide AEC2s with signals to promote self-renewal in addition to preventing apoptosis. We previously collected lung tissue samples from patients with IPF^30^. Through further analysis of the data, we found elevated RNA and protein expression of RAB6 in IPF lung tissues (Supplementary Fig. 1A, B). To further test whether RAB6 is involved in the development of IPF, the expression of RAB6 in PM2.5-induced pulmonary fibrosis in mice was determined. First, we observed that mice were more sensitive to PM2.5-induced damage than saline treatment (Supplementary Fig. 1C), and the alveolar structure of the mice was impaired with a large amount of collagen deposition after exposure to PM2.5 (Supplementary Fig. 1D, E). To determine whether PM2.5 exposure stimulated inflammatory responses and cell apoptosis in mice, the levels of inflammatory factor in alveolar lavage fluid and the alveolar apoptosis were measured by ELISA and TUNEL analysis. We found the levels of IL-1β and TNF-α in alveolar lavage fluid were significantly elevated (Supplementary Fig. 1G). In addition, PM2.5 induced pulmonary fibrosis formation with a large number of alveolar epithelial cell apoptosis (Supplementary Fig. 1F). More importantly, we showed that the expression of RAB6 mRNA and protein was significantly upregulated in mice with lung fibrosis (Supplementary Fig. 1H, I). These results indicated that RAB6 was involved in the development of PM2.5-induced lung injury and pulmonary fibrosis in mice.

**Fig. 1:**
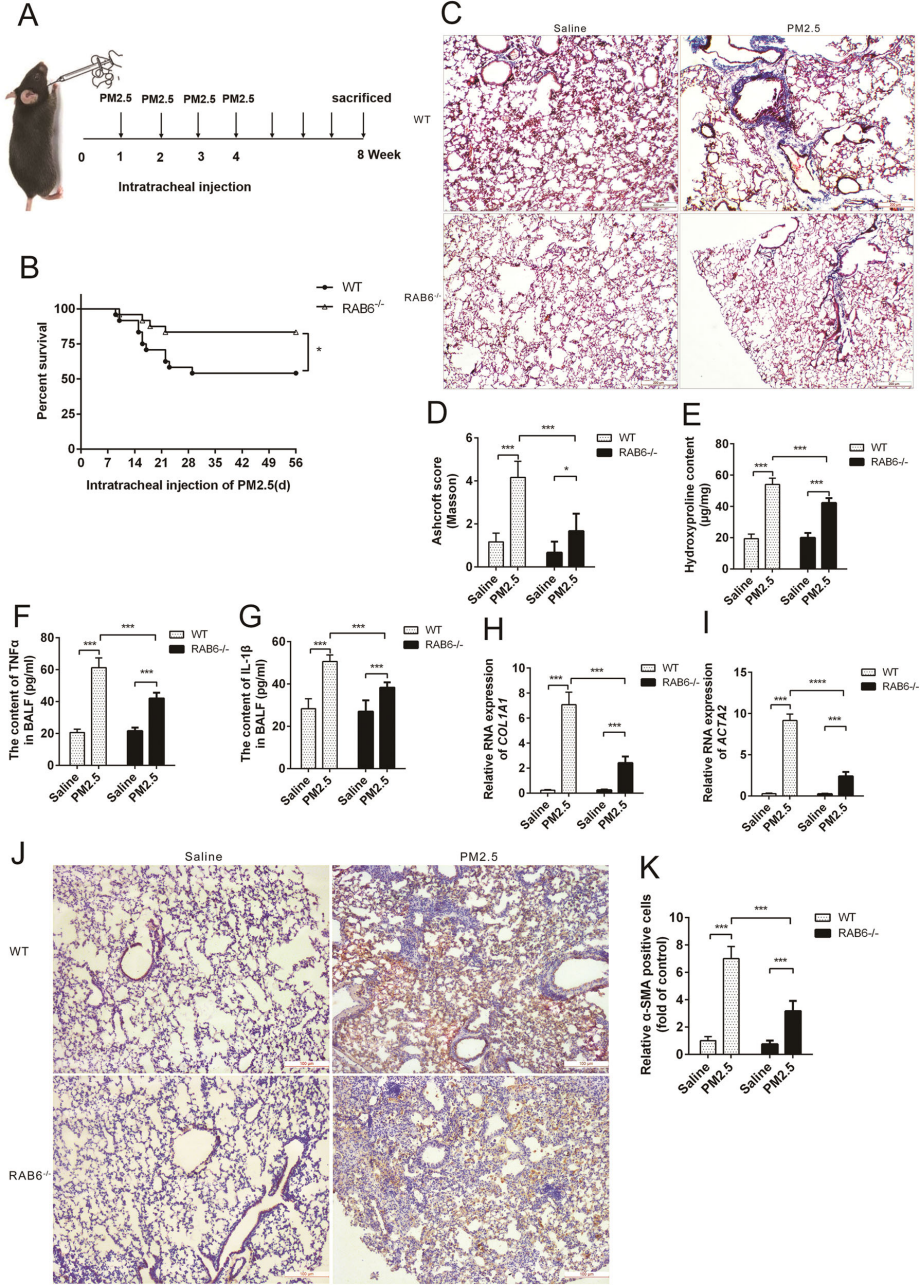
RAB6 deficiency attenuated PM2.5-induced lung injury and fibrosis. **a** Schematic diagram showing the detection of lung injury and fibrosis in mice after PM2.5 intratracheal administration (instilled once a week, and continuously instilled four times) after 8 weeks. **b** Percentages of surviving WT mice (*n* = 24) and RAB6^−/−^ mice (*n* = 24) plotted over a 8-week period after intratracheal treatment with PM2.5. *P < 0.05 by log-rank test. **c** WT and RAB6^−/−^ mice were intratracheally instilled with 50 μl of PM2.5 (100 mg/kg) or saline (instilled once a week, and continuously instilled four times). Lungs were subjected to MASSON staining (Represented micrographs from six mice per group are shown, Scale bar = 200 μm). **d** Quantitative analysis (Ashcroft score) of lung tissue fibrosis in mice. **e** Lung collagen content was determined by hydroxyproline assay. **f**, **g** The content of TNFα (**f**) and IL-1β (**g**) in bronchoalveolar lavage fluid (BALF) were determined by ELISA. **h**, **i** The relative expression of collagen RNA (**h**) and α-SMA RNA (**i**) in WT and RAB6^−/−^ mice exposed to PM2.5 or saline was measured by qRT-PCR. **j** The expression of α-SMA protein in lung tissue of WT and RAB6^−/−^ mice exposed to PM2.5 or saline was determined by immunohistochemistry (Representative micrograph of six individual subjects. Scale bar = 100 μm). **k** Quantification of α-SMA expression in mice lung tissue. (*n* = 6; Unpaired two-tailed *t* test. *P < 0.05; ***P < 0.001. Bar graphs represent the mean ± SEM for **d**–**i**, **k**).

To investigate the role of RAB6 in PM2.5-induced lung injury and fibrosis, we generated mice with total knockout of RAB6 (RAB6^−/−^). The mice were intratracheally instilled with PM2.5 (once per week for 4 weeks), and the lung injury and fibrosis-related test were performed 4 weeks after the last instillation (Fig. 1a). As compared to wild type (WT) mice, RAB6^−/−^ mice were less susceptible to PM2.5-induced lung injury (Fig. 1b), and demonstrated a weakened injury and fibrotic response to PM2.5 as shown by MASSON staining (Fig. 1c, d) and lower hydroxyproline content in lung tissues after PM2.5 exposure (Fig. 1e). We examined the inflammatory response of RAB6^−/−^ mice to PM2.5 lung injury and found that the levels of IL-1β and TNFα in the alveolar fluid of RAB6^−/−^ mice were significantly lower than those in WT mice after PM2.5 exposure (Fig. 1f, g). In addition, We observed that lower fibrosis in RAB6^−/−^ mice was accompanied by decreased expression of α-SMA RNA, collagen RNA and collagen as compared to WT mice (Fig. 1h, i and Supplementary Fig. 2). Moreover, immunohistochemistry assessment showed that α-SMA protein staining in the lung tissue of RAB6^−/−^ mice was significantly lower than that of WT mice after exposure to PM2.5 (Fig. 1j, k). Together, these data suggest that RAB6^−/−^ mice exhibited lower levels of lung inflammation and fibrotic response than WT mice after exposure to PM2.5.

### RAB6 deficiency attenuated lung oxidative stress in PM2.5-injured mice

**Fig. 2:**
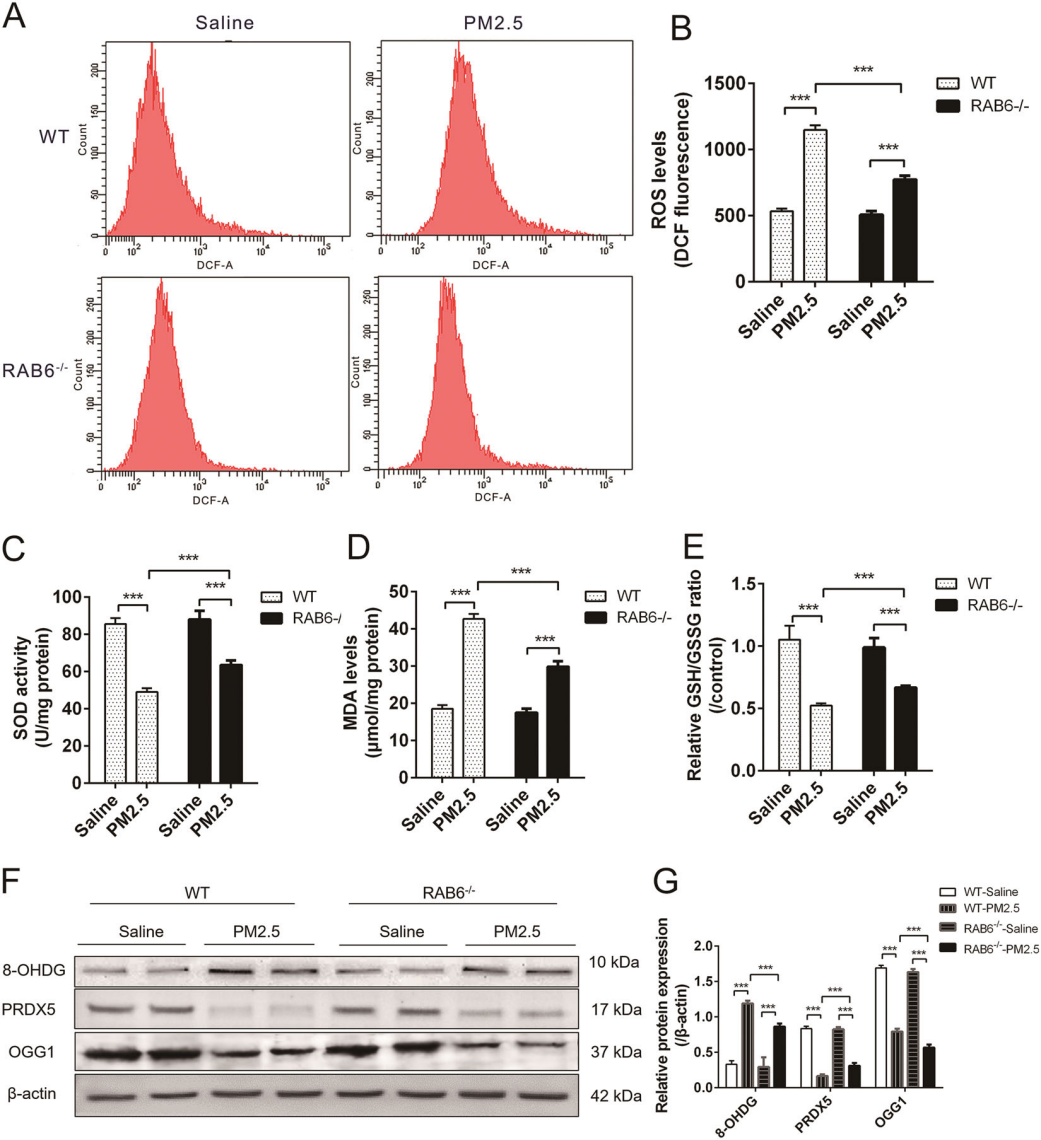
RAB6 deficiency attenuated lung oxidative stress in PM2.5-injured mice. WT and RAB6^−/−^ mice were intratracheally instilled with 50 μl of PM2.5 (100 mg/kg) or saline (instilled once a week, and continuously instilled four times). **a** Lung tissue ROS levels were measured by FACS (DCFH-DA fluorescent probe). (Representative FACS images are shown, *n* = 6) **b** Statistical analysis of ROS levels (DCF fluorescence value). **c–e** The superoxide dismutase (SOD) activity (**c**), malondialdehyde (MDA) content (**d**), and the ratio of reduced glutathione to oxidized glutathione (GSH/GSSG) (**e**) in lung tissue were detected by chemiluminescence analysis. **f** The relative protein expression of 8-OHDG, PRDX5 and OGG1 in lung tissue was detected by western blot analysis. **g** Statistical analysis of relative expression levels of proteins. (*n* = 6; Unpaired two-tailed *t* test. ***P < 0.001. Bar graphs represent the mean ± SEM for **b**, **d**, and **g**).

To examine the mechanism by which RAB6 attenuated PM2.5-induced pulmonary inflammatory responses, we further examined the effect of knockout of RAB6 on PM2.5-induced oxidative stress in lung tissue of mice. The levels of ROS in lung tissue RAB6^−/−^ mice was significantly reduced compared with WT mice after exposure to PM2.5 (Fig. 2a, b). Additionally, after exposure to PM2.5, the antioxidant enzyme SOD activity and GSH/GSSG ratio of lung tissue in WT mice were significantly decreased, and the level of MDA was increased; however, these changes were significantly reduced in RAB6-/- mice (Fig. 2c–e). Since PM2.5-induced oxidative stress can cause mitochondrial dysfunction and DNA oxidative damage in alveolar epithelial cells^8,34^, we examined the expression of cellular antioxidant enzymes and DNA damage marker proteins 8-hydroxy-2′-deoxyguanosine (8-OHDG) by western blot. The expression of DNA base excision repair enzyme (OGG1) and peroxiredoxin (PRDX5) were significantly decreased, and the expression of 8-OHDG increased in WT mice after exposure to PM2.5. In contrast, the expression of PRDX5 and OGG1 in the lung tissue of RAB6^−/−^ mice were significantly increased, and the expression of 8-OHDG was reduced as compared with WT mice after exposure to PM2.5 (Fig. 2f, g). These data demonstrate that RAB6 deficiency attenuates lung oxidative stress in PM2.5-exposed mice.

### RAB6 deficiency attenuated alveolar epithelial cell death in PM2.5-injured mice

**Fig. 3:**
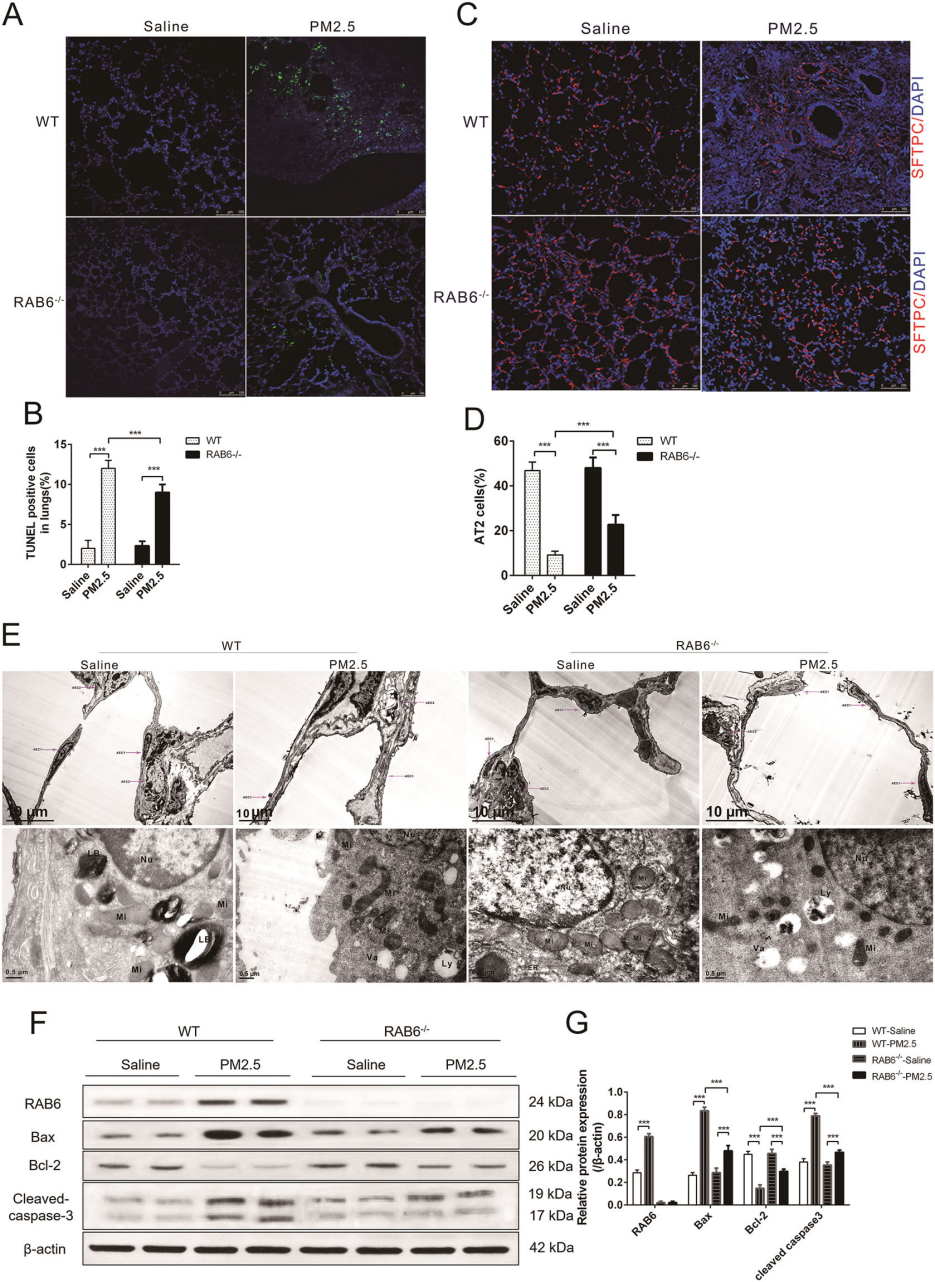
RAB6 deficiency attenuated alveolar epithelial cell death in PM2.5-injured mice. **a** WT and RAB6^−/−^ mice were intratracheally instilled with 50 μl of PM2.5 (100 mg/kg) or saline (instilled once a week, and continuously instilled four times). The apoptosis of alveolar epithelial cells in mice was determined by TUNEL staining (represented micrographs from six mice per group are shown, Scale bar = 100 μm). **b** Quantitative analysis of TUNEL positive cells. **c** The SFTPC^+^ AEC2 cells in lung tissue was detected by immunofluorescence staining (Blue, DAPI; red, SFTPC). (Represented micrographs from 6 mice per group are shown, Scale bar = 100 μm). **d** Quantitative analysis of the proportion of SFTPC^+^ AEC2 cells in lung tissue. **e** The ultrastructure of AEC1 cells and AEC2 cells in lung tissue was detected by transmission electron microscopy (TEM) (Nu nucleus; Mi mitochondrion; LB lamellar body; Ly lysosome; Va vacuoles) (Upper panel, scale bar = 10 μm; lower panel, scale bar = 0.5 μm). **f** The relative protein expression of RAB6, Bax, Bcl-2, and cleaved-caspase-3 in lung tissue was detected by western blot analysis. **g** Statistical analysis of relative expression levels of proteins. (*n* = 6; unpaired two-tailed *t* test. ***P < 0.001. Bar graphs represent the mean ± SEM for **b**, **d**, and **g**).

We first examined the effect of RAB6 on PM2.5-induced apoptosis in alveolar epithelial cells. As shown by the TUNEL results, the number of apoptotic cells in RAB6^−/−^ mice was significantly lower than that in WT mice (Fig. 3a, b). We, then, further examined the effect of RAB6 on SFTPC^+^AEC2 cells in lung tissue. We found there was no significant difference in the number of AEC2 cells in the lungs of uninjured RAB6^−/−^ mice and WT mice, but the number of SFTPC^+^AEC2 cells in the lung tissue of PM2.5-injured RAB6^−/−^ mice was significantly greater than that in the PM2.5-injured WT mice (Fig. 3c, d). Moreover, TEM was used to evaluate the effect of RAB6 on the ultrastructure of lung tissue in mice with PM2.5 injury. We observed that PM2.5 injury caused swelling of mitochondria and the appearance of lysosomes in mouse AEC2 cells. The swelled mitochondria in AEC2 cells decreased in RAB6^−/−^ mice as compared to WT mice (Fig. 3e). Furthermore, We examined the expression of mitochondrial apoptosis-associated proteins by immunoblotting, and found that the expression of Bcl-2 in lung tissue of PM2.5-injured RAB6^−/−^ mice was higher while the expression of caspase-3 and BAX was lower (Fig. 3f, g) than those in the PM2.5-injured WT mice. Together, these data suggest that RAB6 deficiency attenuates alveolar epithelial cell death in PM2.5-injured mice

### Knockout of RAB6 promoted the self-renewal and proliferation of AEC2s

**Fig. 4:**
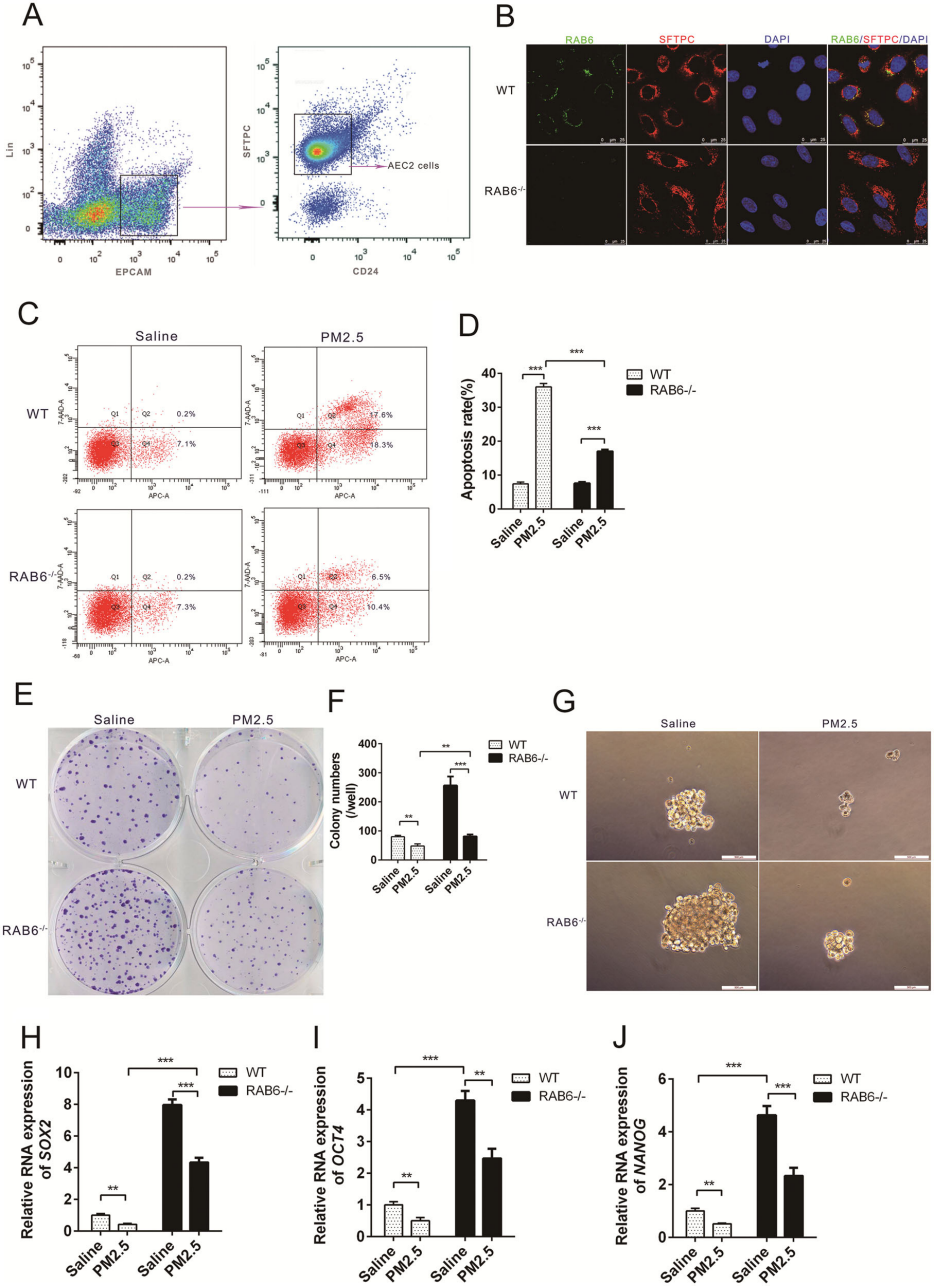
Knockout of RAB6 promoted the self-renewal and proliferation of AEC2s. **a** The enriched AEC2 cell population was isolated by selection of the CD24^−^ SFTPC^+^ subset from epithelial cell populations (EpCAM^+^CD31^−^CD34^−^CD45^−^) in lung tissues by FACS. **b** The localization and expression of RAB6 and SFTPC proteins in WT and RAB6^−/−^ AEC2 cells were detected by immunofluorescence (Green, RAB6; red, SFTPC; blue, DAPI) (Representative immunofluorescence images are shown, *n* = 3). **c** WT and RAB6^−/−^ AEC2 cells were exposed to PM2.5 (100 μg/ml) or saline for 48 h. The apoptosis of AEC2 cells was determined by FACS (Representative FACS images are shown, n = 3). **d** Statistical analysis of apoptosis rate. **e** The colony forming efficiency (CFE) of WT and RAB6^−/−^ AEC2 cells was examined by plate colony formation assay (Representative images are shown, *n* = 3). **f** Statistical analysis of CFE (represented by colony numbers). **g** Self-renewal (spherication ability) of WT and RAB6^−/−^ AEC2 cells exposed to PM2.5 or saline was detected by 3D culture (Representative images are shown, *n* = 3). **h**–**j** The relative RNA expression of SOX2 (**h**), OCT4 (**i**), and NANOG (**j**) was measured by qRT-PCR. (*n* = 3; unpaired two-tailed *t* test. **P < 0.01; ***P < 0.001. Bar graphs represent the mean ± SEM for **d**, **f**, **h**–**j**).

We used mouse lung tissue dissociation and fractionation methods to generate alveolar epithelial cell populations (EPCAM^+^ Lin^−^), and the enriched AEC2 cell population was isolated by selection of the CD24^−^ SFTPC^+^ subset from epithelial cell populations (Fig. 4a). We observed that the sorted AEC2 cells had a lamellar body structure (Supplementary Fig. 3A), and flow cytometry showed that they were SFTPC positive cells (Supplementary Fig. 3B). The expression of pro-SPC protein was up-regulated in sorted AEC2 cells (Supplementary Fig. 3C, D). Moreover, flow cytometry sorting of AEC2 cells enriched for expression of SFTPA1, SFTPB, and ABCA3 transcripts (Supplementary Fig. 3E, F, G). We observed that there was no significant change in the expression of pro-SPC protein in AEC2 cells cultured for 1 week under the same experimental conditions (Supplementary Fig. 4A, B). In addition, there was no significant change in the RNA expression of SFTPA1, SFTPB, and ABCA3 transcripts in AEC2 cells cultured for 1 week (Supplementary Fig. 4C–E). The expression of RAB6 and SFTPC in isolated AEC2 cells (cultured for more than 1 week) from WT and RAB6^−/−^ mice was further verified by immunofluorescence (Fig. 4b). Flow cytometry showed that the apoptosis rate of PM2.5-injured RAB6^−/−^ AEC2 cells was lower than that of PM2.5-injured WT AEC2 cells (Fig. 4c, d). Moreover, we examined the effect of knocking out RAB6 on colony forming ability of PM2.5-injured AEC2s. We observed an increase in the number colonies of PM2.5-injured AEC2 cells after knocking out of RAB6 (Fig. 4e, f). In addition, RAB6^−/−^ AEC2 cells showed an increase in sphere size as compared with WT AEC2 cells (Fig. 4g). Finally, we examined the effect of RAB6 knock-out on the expression of stem cell pluripotency transcription factors (SOX2, OCT4, and NANOG). The results showed that the expression of SOX2, OCT4, and NANOG were significantly increased in RAB6^−/−^ AEC2 cells as compared with WT AEC2 cells (Fig. 4h–j). These data indicate that knockout of RAB6 promotes the self-renewal and proliferation of AEC2 cells in vitro.

### Knockout of RAB6 inhibited DKK1 autocrine and activated wnt/β-catenin signaling in PM2.5-injured AEC2 cells

**Fig. 5:**
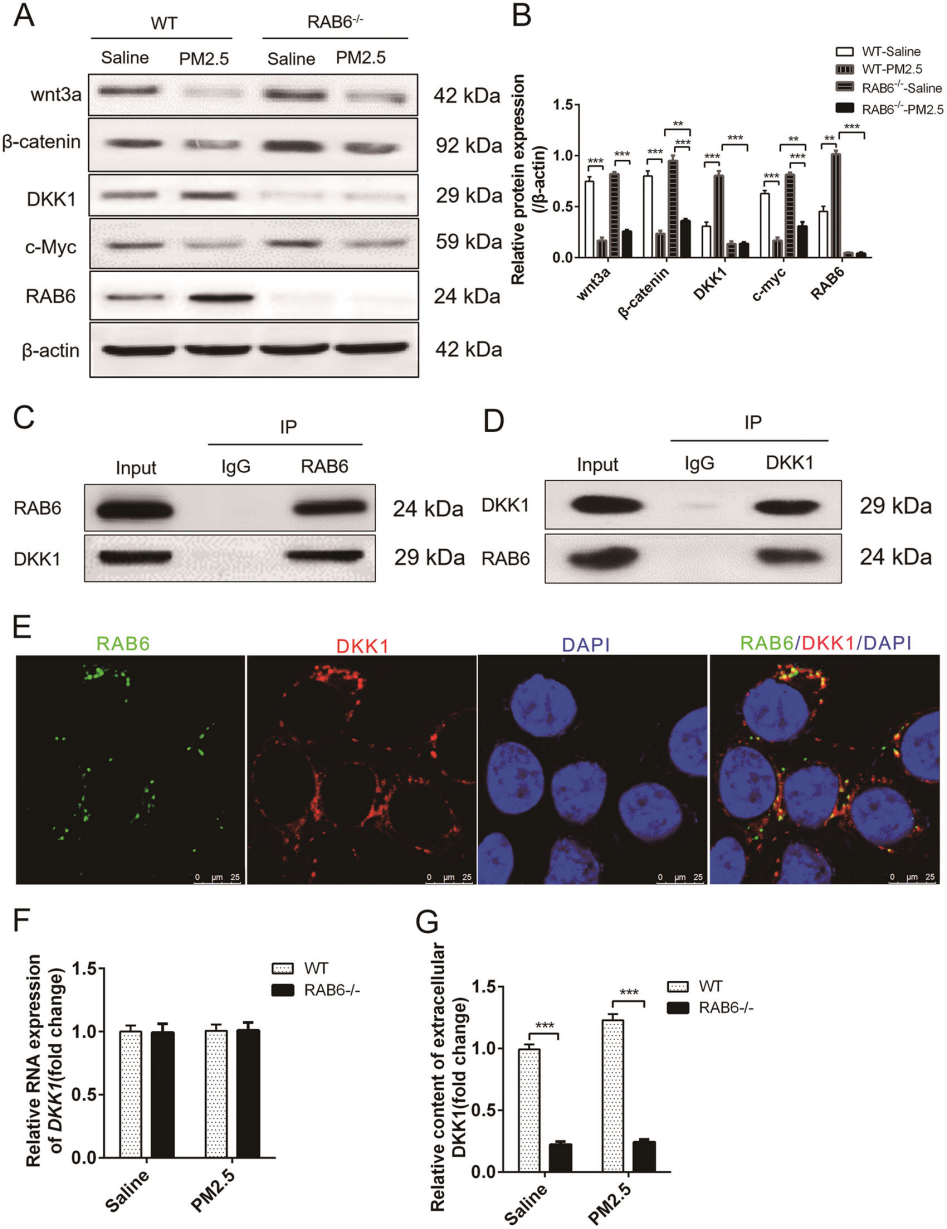
Knockout of RAB6 inhibited DKK1 autocrine and activated wnt/β-catenin signaling in PM2.5-injured AEC2 cells. **a** WT and RAB6^−/−^ AEC2 cells were exposed to PM2.5 (100 μg/ml) or saline for 48 h. The relative protein expression of wnt3a, β-catenin, DKK1, c-myc, and RAB6 in AEC2 cells was detected by western blot analysis. **b** Statistical analysis of relative expression levels of proteins. **c**, **d** The interaction between endogenous RAB6 and DKK1 in WT AEC2 cells was detected by Co-Immunoprecipitation (Co-IP) assay (IgG, negative control; Input, cell lysate). **e** The co-localization and expression of RAB6 and DKK1 proteins in WT AEC2 cells were detected by immunofluorescence (Green, RAB6; red, DKK1; blue, DAPI) (Representative immunofluorescence images are shown, *n* = 3). **f** The relative expression of DKK1 RNA in WT and RAB6^−/−^ AEC2 cells exposed to PM2.5 or saline was measured by qRT-PCR. **g** The content DKK1 protein secreted into extracellular culture medium by AEC2 cells was detected by ELISA. (*n* = 3; unpaired two-tailed *t* test. **P < 0.01; ***P < 0.001. Bar graphs represent the mean ± SEM for **b**, **f**, and **g**).

We have previously observed that the wnt/β-catenin pathway regulated the EMT process of AEC2 cells that are involved in the development of pulmonary fibrosis, which prompted us to look for factors in the wnt/β-catenin pathway that may be involved in RAB6 regulation of AEC2 cell proliferation and self-renewal^30^. As one of the most important regulators of wnt signaling, Dickkopf1 (DKK1) not only regulates stem cell characteristics, but also affects the proliferation of lung epithelial cells in IPF^35,36^. After exposure to PM2.5, the expression of wnt3a, β-catenin, and c-myc was decreased in AEC2 cells, and the expression of RAB6 and DKK1 was increased. In addition, we observed an increase in the expression of wnt3a, β-catenin, and c-myc in PM2.5-injured AEC2 cells after knocking out of RAB6. However, the protein expression of RAB6 and DKK1 was hardly detectable in RAB6^−/−^ AEC2 cells (Fig. 5a, b). Based on the consistent changes in DKK1 and RAB6 proteins, we further examined the presence of interactions between the two proteins. The DKK1 protein was identified in the protein complex precipitated by the RAB6 antibody in WT AEC2 cell lysate by Co-IP, and the RAB6 protein was also identified in the protein complex precipitated by the DKK1 antibody (Fig. 5c, d). Moreover, immunofluorescence results showed that DKK1 and RAB6 proteins were co-localized in the cytoplasm and membrane of WT AEC2 cells (Fig. 5e). Interestingly, the knockout of RAB6 did not inhibit the RNA level of DKK1 as expected (Fig. 5f). More importantly, the knockout of RAB6 suppressed the protein content of extracellular DKK1 (Fig. 5g). These results indicate that knockout of RAB6 inhibits the secretion of DKK1 and activates the wnt/β-catenin signaling.

### DKK1 inhibited proliferation and self-renewal of RAB6^−/−^ AEC2 cells

**Fig. 6:**
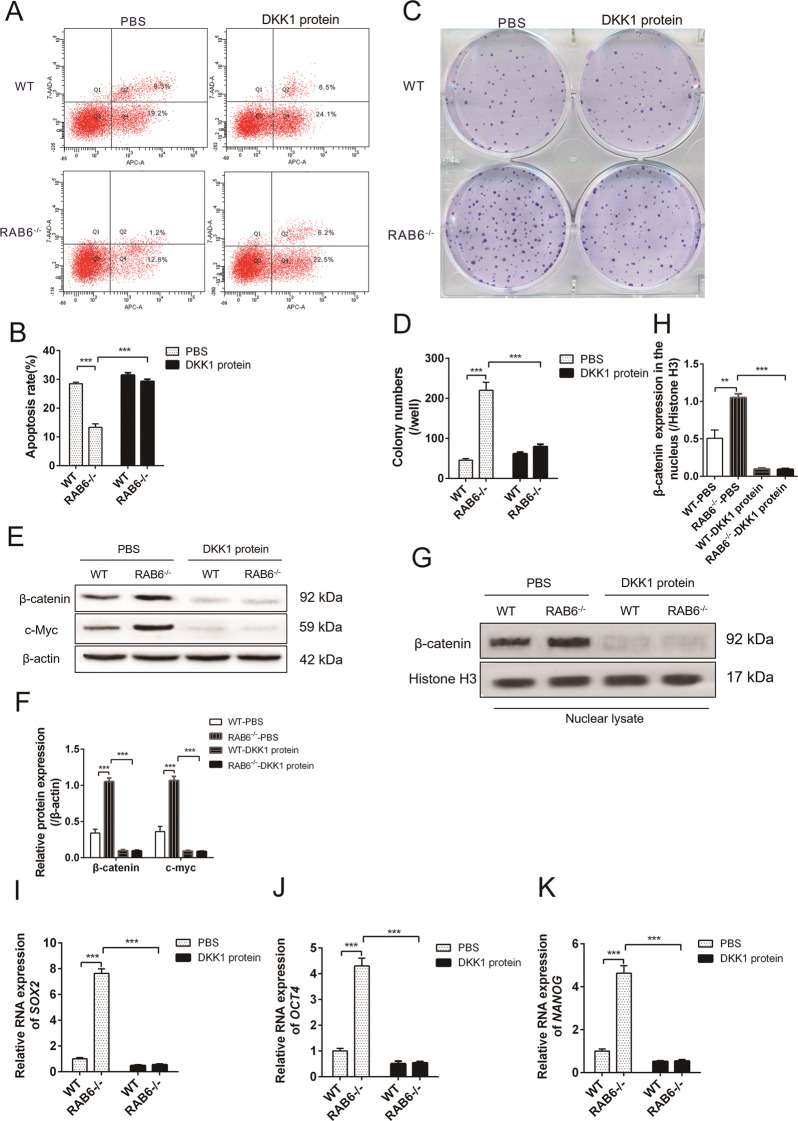
DKK1 inhibited proliferation and self-renewal of RAB6-/- AEC2 cells. **a** WT and RAB6^−/−^ AEC2 cells were exposed to DKK1 protein (10 ng/ml) or PBS for 48 h and then cells were exposed to PM2.5 (100 μg/ml) for 48 h. The apoptosis of AEC2 cells was determined by FACS (Representative FACS images are shown, *n* = 3). **b** Statistical analysis of apoptosis rate. **c** The colony forming efficiency (CFE) of WT and RAB6^−/−^ AEC2 cells was examined by plate colony formation assay (Representative images are shown, *n* = 3). **d** Statistical analysis of CFE (represented by colony numbers). **e** The relative protein expression of β-catenin, and c-myc in AEC2 cell lysates was detected by western blot analysis. **f** Statistical analysis of relative expression levels of proteins. **g** The relative protein expression of β-catenin in nuclear lysates of AEC2 cells was detected by western blot analysis. **h** Statistical analysis of relative expression levels of proteins. **i–k** The relative RNA expression of SOX2 (**i**), OCT4 (**j**), and NANOG (k) was measured by qRT-PCR. (*n* = 3; unpaired two-tailed *t* test. **P < 0.01; ***P < 0.001. Bar graphs represent the mean ± SEM for **b**, **d**, **f**, **h**–**k**).

To test the hypothesis that impaired secretion of DKK1 may contribute to the enhancement of self-renewal and proliferation ability of RAB6^−/−^ AEC2 cells, RAB6^−/−^ AEC2 cells were treated with recombinant DKK1 protein. We observed that overexpression of DKK1 protein abolished the anti-apoptotic effect of RAB6^−/−^AEC2 cells on PM2.5 injury (Fig. 6a, b). In addition, DKK1 protein also inhibited the colony formation of RAB6^−/−^ AEC2 cells (Fig. 6c, d). Furthermore, DKK1 protein inhibited the expression of β-catenin and c-myc proteins in RAB6^−/−^ AEC2 cells, and also inhibited the level of β-catenin in the nucleus (Fig. 6e–h). Finally, we observed a significant decrease in the RNA levels of SOX2, OCT4 and NANOG in the RAB6^−/−^ AEC2 cells treated with DKK1 protein (Fig. 6i–k). Taken together, these data shown that RAB6 regulates proliferation and self-renewal of AEC2 cells via DKK1.

### RAB6 inhibited wnt signaling, proliferation, and self-renewal of AEC2 cells by promoting DKK1 autocrine

**Fig. 7:**
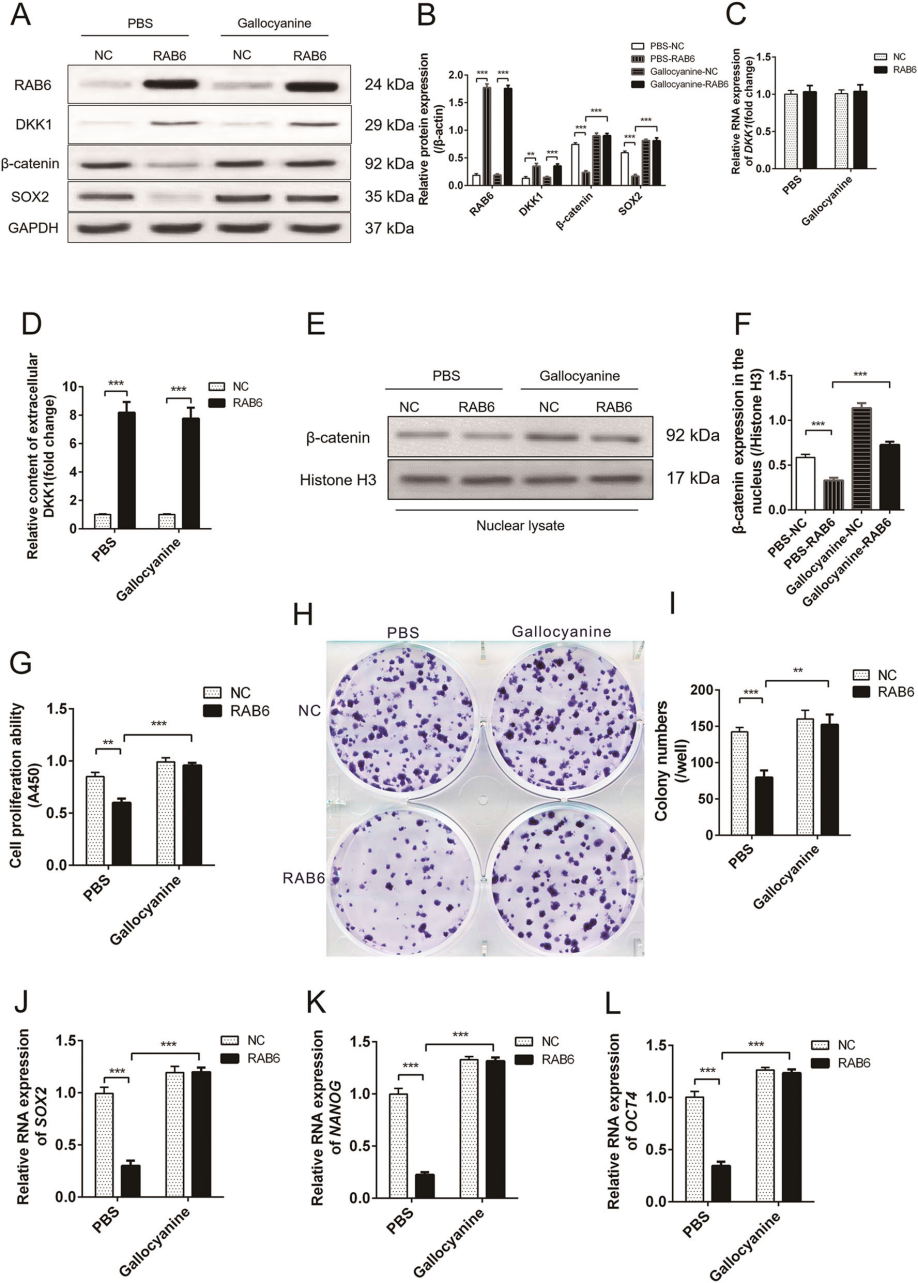
RAB6 inhibited wnt signaling, proliferation, and self-renewal of AEC2 cells by promoting DKK1 autocrine. **a** RAB6 overexpression (RAB6) and negative control (NC) AEC2 cells were exposed to DKK1 inhibitor (Gallocyanine, 5 μM) or PBS for 48 h. The relative protein expression of RAB6, DKK1, β-catenin and SOX2 in AEC2 cells was detected by western blot analysis. **b** Statistical analysis of relative expression levels of proteins. **c** The relative expression of DKK1 RNA in NC and RAB6 AEC2 cells exposed to Gallocyanine or PBS was measured by qRT-PCR. **d** The content DKK1 protein secreted into extracellular culture medium by AEC2 cells was detected by ELISA. **e** The relative protein expression of β-catenin in nuclear lysates of AEC2 cells was detected by Western blot analysis. **h** Statistical analysis of relative expression levels of proteins. **g** The proliferation of NC and RAB6 AEC2 cells exposed to Gallocyanine or PBS was measured by CCK8 assay. (**h**) The colony forming efficiency (CFE) of NC and RAB6 AEC2 cells was examined by plate colony formation assay (Representative images are shown, *n* = 3). **i** Statistical analysis of CFE (represented by colony numbers). **j**–**l** The relative RNA expression of SOX2 (**j**), OCT4 (**k**) and NANOG (**l**) was measured by qRT-PCR. (*n* = 3; unpaired two-tailed *t* test. **P < 0.01; ***P < 0.001. Bar graphs represent the mean ± SEM for **b**–**d**, **f**, **g**, **i**–**l**).

To test the hypothesis that increased secretion of DKK1 leads to a decrease in self-renewal and proliferation of RAB6 overexpressing AEC2 cells, DKK1 inhibitor-treated RAB6 overexpressing AEC2 cells were evaluated. We observed that DKK1 inhibitor-Gallocyanine promoted the expression of β-catenin and SOX2 proteins in RAB6 overexpressing AEC2 cells and promoted the level of β-catenin in the nucleus (Fig. 7a, b, e, f), but had no effect on DKK1 RNA levels and autocrine(Fig. 7c, d). This indicates that the inhibitor does not affect the synthesis and autocrine of DKK1, but inhibits the interaction of DKK1 with the receptor. In addition, Gallocyanine also promoted the proliferation and CFE of RAB6 overexpressing AEC2 cells (Fig. 7g–i). Furthermore, we observed a significant increase in the RNA levels of SOX2, OCT4 and NANOG in RAB6 overexpressing AEC2 cells treated with Gallocyanine (Fig. 7j–l). These data further confirm that RAB6 regulates proliferation and self-renewal of AEC2 cells by modulating the autocrine of DKK1.

### Inhibition of DKK1 attenuated PM2.5-induced pulmonary fibrosis in vivo

**Fig. 8:**
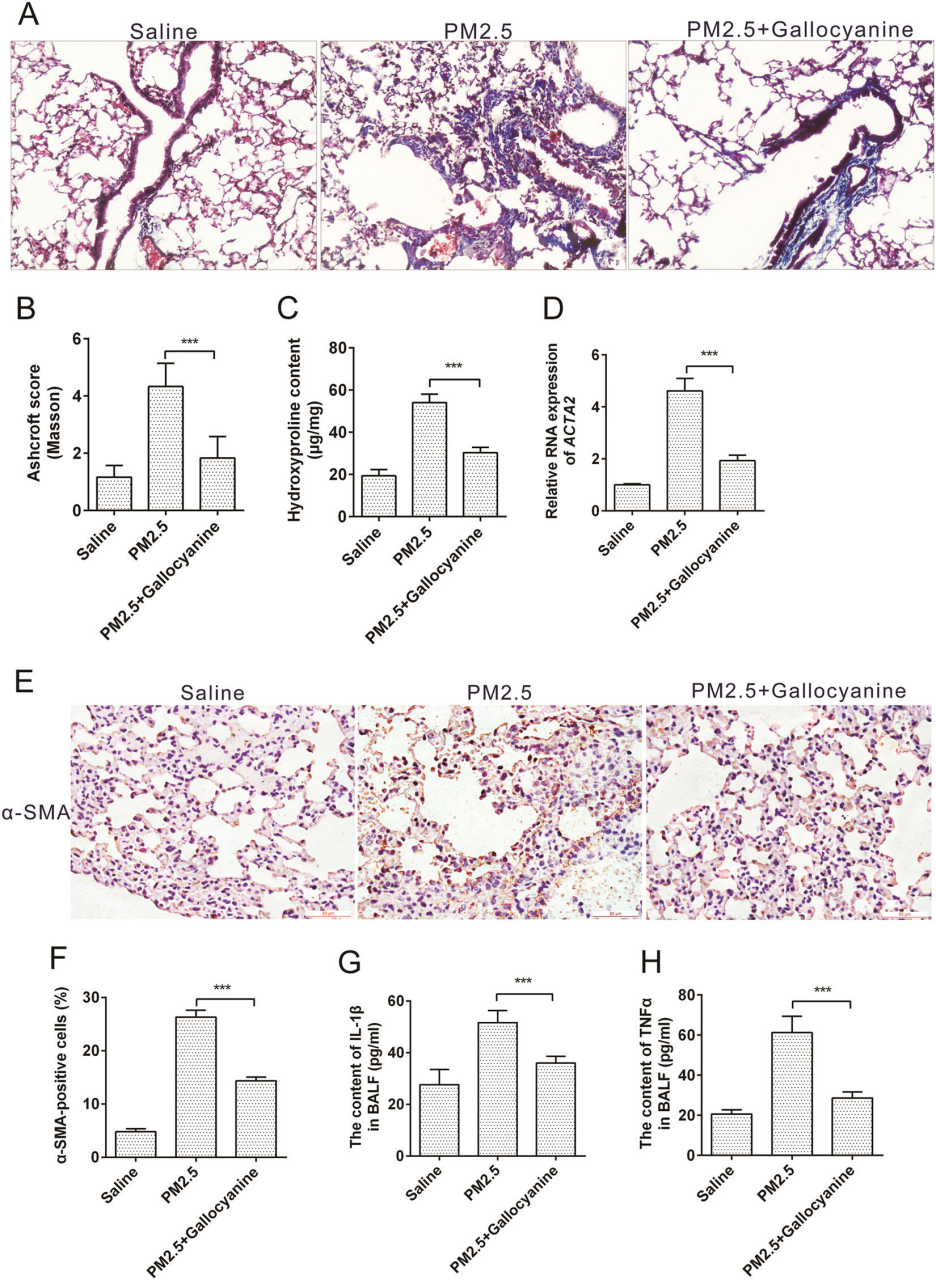
Inhibition of DKK1 attenuated PM2.5-induced pulmonary fibrosis in vivo. **a** WT C57BL/6J mice were intratracheally instilled with 50 μl of saline, PM2.5 (100 mg/kg) or PM2.5+ Gallocyanine(100 mg/kg + 1 mg/kg) (instilled once a week, and continuously instilled four times). Lungs were subjected to MASSON staining (Represented micrographs from six mice per group are shown, Scale bar = 200 μm). **b** Quantitative analysis (Ashcroft score) of lung tissue fibrosis in mice. **c** Lung collagen content was determined by hydroxyproline assay. **d** The relative expression of α-SMA RNA in mouse lung tissues was measured by qRT-PCR. **e**, **f** The expression of α-SMA protein in lung tissue of mice exposed to saline, PM2.5 or PM2.5+ Gallocyanine was determined by immunohistochemistry (Representative micrograph of six individual subjects. Scale bar = 50 μm). **g**, **h** The content of IL-1β (**g**) and TNFα (**h**) in bronchoalveolar lavage fluid (BALF) were determined by ELISA. (*n* = 6; unpaired two-tailed *t* test. ***P < 0.001. Bar graphs represent the mean ± SEM for **b**–**d**, **f**–**h**).

The observation that RAB6^−/−^ AEC2s with high colony formation and self-renewal ability was associated with lower expression of DKK1 prompted us to investigate the effect of DKK1 inhibitor on PM2.5-induced pulmonary fibrosis in vivo. Similar to what we observed in RAB6^−/−^ mice, mice treated with a small molecule DKK1 inhibitor-Gallocyanine demonstrated an ameliorated injury and fibrotic response to PM2.5 as shown by MASSON staining (Fig. 8a, b) and lower hydroxyproline content in lung tissues after PM2.5 injury (Fig. 8c). In addition, we observed a decrease in RNA levels of a-SMA (Fig. 8d) and protein levels of a-SMA (Fig. 8e, f) in lung tissue of Gallocyanine-treated PM2.5-injured mice. Finally, we examined the inflammatory response of Gallocyanine-treated mice to PM2.5 lung injury and observed that the levels of IL-1β and TNFα in the alveolar fluid of Gallocyanine-treated PM2.5-injured mice were decreased (Fig. 8g, h). Overall, these finding shown that DKK1 inhibitor attenuates PM2.5-induced pulmonary fibrosis in mice.

## Discussion

One of the key issues in the pathology of pulmonary fibrosis is the lack of understanding of the molecular mechanisms underlying AEC2 proliferation and self-renewal after injury. The major finding of our current study is that RAB6 deficiency attenuates PM2.5-induced lung injury and fibrosis in mice by inhibiting alveolar epithelial cell death and lung oxidative stress in PM2.5-injured mice. In addition, consistent with in vivo results, knockout of RAB6 promoted self-renewal and proliferation of AEC2 in vitro. More importantly, RAB6 regulates wnt/β-catenin signaling by interacting with DKK1 protein to regulate the proliferation and self-renewal of AEC2 cells. Moreover, inhibition of DKK1 in vivo can alleviate PM2.5-induced pulmonary inflammation and fibrosis.

It is well known that the lung is the direct target organ for inhaling damage caused by PM2.5. Accumulating data suggest that the increasing PM2.5 is associated with the development of various lung diseases including asthma, lung injury, and pulmonary fibrosis^10,37,38^. PM2.5 mainly contains 16 kinds of polycyclic aromatic hydrocarbons (PAHs) and metals, and excessive production of ROS is one of the key factors for PM2.5-induced cell damage. Our previous studies have shown that PM2.5 aggravates allergic asthma in mice by promoting inflammatory responses and inhibiting autophagy^39^. At the same time, we also found that PM2.5 promotes apoptosis and autophagy in alveolar epithelial cells through ROS/AMPK signaling^8^. However, the underlying mechanisms of PM2.5 effects on AEC2 cell self-renewal and pulmonary fibrosis have not been elucidated. In this study, we show that continuous PM2.5 stimulation induces oxidative stress in the lungs, impairs AEC2 cell proliferation and self-renewal, and promotes pulmonary fibrosis in mice. These changes can be alleviated by knocking out RAB6. Our observations support the notion that IPF is primarily a disease of AEC2 stem cell failure.

AEC2 cells, as progenitor cells of the lung, undergo self-renewal and trans-differentiation after birth, thereby giving rise to AEC1 cells during homeostatic processes or after tissue damage^40^. Signaling pathways that promote proliferation and self-renewal of damaged AEC2 cells during alveolar repair are incompletely understood. Our previous studies showed that down-regulation of RAB6 contributes to the enhancement of self-renewal of LCSCs^29^. In this study, we tested the hypothesis that RAB6 is involved in the regulation of proliferation and renewal of impaired AEC2s during alveolar repair. We show that AEC2 cells lacking RAB6 have higher proliferation and self-renewal capacity and activated wnt/β-catenin signaling in vitro and lead to less PM2.5-induced lung damage in vivo. In addition, overexpression of RAB6 causes a lower proliferation and self-renewal ability of AEC2 cells in vitro and inhibition of wnt/β-catenin signaling. However, it is unclear whether the expression of AEC2 cell-specific proteins and transcription factors will change after PM2.5 stimulation.

Canonical Wnt/β-catenin signaling pathway is involved in stem cell proliferation and self-renewal regulation in many tissues^40^. Stem cell populations in various tissues have been identified by Wnt signaling activity. Wnt/β-catenin signaling is the major regulator of stemness gene activity including SOX2, OCT4, and NANOG^41–43^. Our previous studies have shown that the wnt/β-catenin signaling pathway in AEC2 cells is involved in the development of pulmonary fibrosis by regulating epithelial-mesenchymal transition (EMT)^30^. In this report, we demonstrate that the deletion of RAB6 increases proliferation and self-renewal of AEC2 cells, accompanied by activation of Wnt signaling and its downstream stemness genes. Moreover, the wnt inhibitor-DKK1 eliminates the increase in proliferation and self-renewal caused by knockout of RAB6. These findings support the notion that RAB6 deficiency promotes AEC2 cell proliferation and self-renewal by activating wnt signaling.

A provocative finding in this study is that RAB6 regulates the wnt/β-catenin signal by interacting with DKK1 and regulating the secretion of DKK1 protein. Upstream signals that regulate wnt/β-catenin include extracellular wnt proteins and secreted Dickkopf (DKK) and SFRPs^44^. Among these DKK proteins, DKK1 has been demonstrated to be effective antagonists of canonical Wnt signaling by directly binding to Wnt coreceptor LRP 5/6 with high affinities^45^. The regulation of DKK1 by RAB6 is intriguing. In this study, we demonstrate that RAB6 interacts with DKK1 in WT AEC2 cells. In addition, RAB6 and DKK1 proteins were co-localized in the cytoplasm and membrane of WT AEC2 cells. We show that knockdown of RAB6 does not affect DKK1 RNA levels, but inhibits extracellular DKK1 protein levels. This indicates that RAB6 regulates the modification, transport, and secretion of DKK1 protein after translation. Moreover, increased extracellular DKK1 protein inhibits/reverts the effect of RBA6 knockdown on AEC2 cells, and DKK1 antagonist inhibits the effect of RBA6 overexpression on wnt/β-catenin signaling and AEC2 cell proliferation and self-renewal. This further confirms that RAB6 affects AEC2 cell proliferation and self-renewal by regulating the autocrine of DKK1 and wnt/β-catenin signaling. Despite this, the elucidation of the specific molecular mechanism by which RAB6 regulates DKK1 autocrine still requires further investigation. Finally, we cannot completely rule out the possibility that RAB6 regulates AEC2 cell proliferation and self-renewal through other potential targets, such as cell division control protein 42 (CDC42)^25^ and kinesin-1^46^, all of which were confirmed as targets of RAB6 by other investigators.

In summary, our data support the notion that IPF is primarily a disease of AEC2 stem cell failure. The data in this report provided a strong molecular link between RAB6, PM2.5 injury, and self-renewal of AEC2 cells. We show that RAB6 deficiency inhibits alveolar epithelial cell death, reduces lung inflammation and oxidative stress to inhibit PM2.5-induced lung injury and fibrosis. In addition, RAB6 interacts with the wnt signaling inhibitor-DKK1 protein, and knockout of RAB6 activates wnt/β-catenin signaling by targeting inhibition of DKK1 autocrine, thereby promoting proliferation and self-renewal of AEC2 cells (Supplementary Fig. 5). The roles of RAB6/DKK1 axis in AEC2 cells gives credence to support that inhibition of RAB6 to subsequently enhance wnt/β-catenin signal as a potential strategy to target IPF.

## Supplementary information

Supplementary Figure 1

Supplementary Figure 2

Supplementary Figure 3

Supplementary Figure 4

Supplementary Figure 5

Supplementary Figure Legends

Supplementary Table 1

Supplementary Table 2

Supplementary Table 3

## Data Availability

The data used to support the findings of this study are available from the corresponding author upon request.
