# Supplementary figures and images for: Small GTPase RAB6 deficiency promotes alveolar progenitor cell renewal and attenuates PM2.5-induced lung injury and fibrosis
Source: Cell Death Dis. 2020 Oct 4;11(10):827. doi: 10.1038/s41419-020-03027-2 (PMC7533251; doi:10.1038/s41419-020-03027-2)

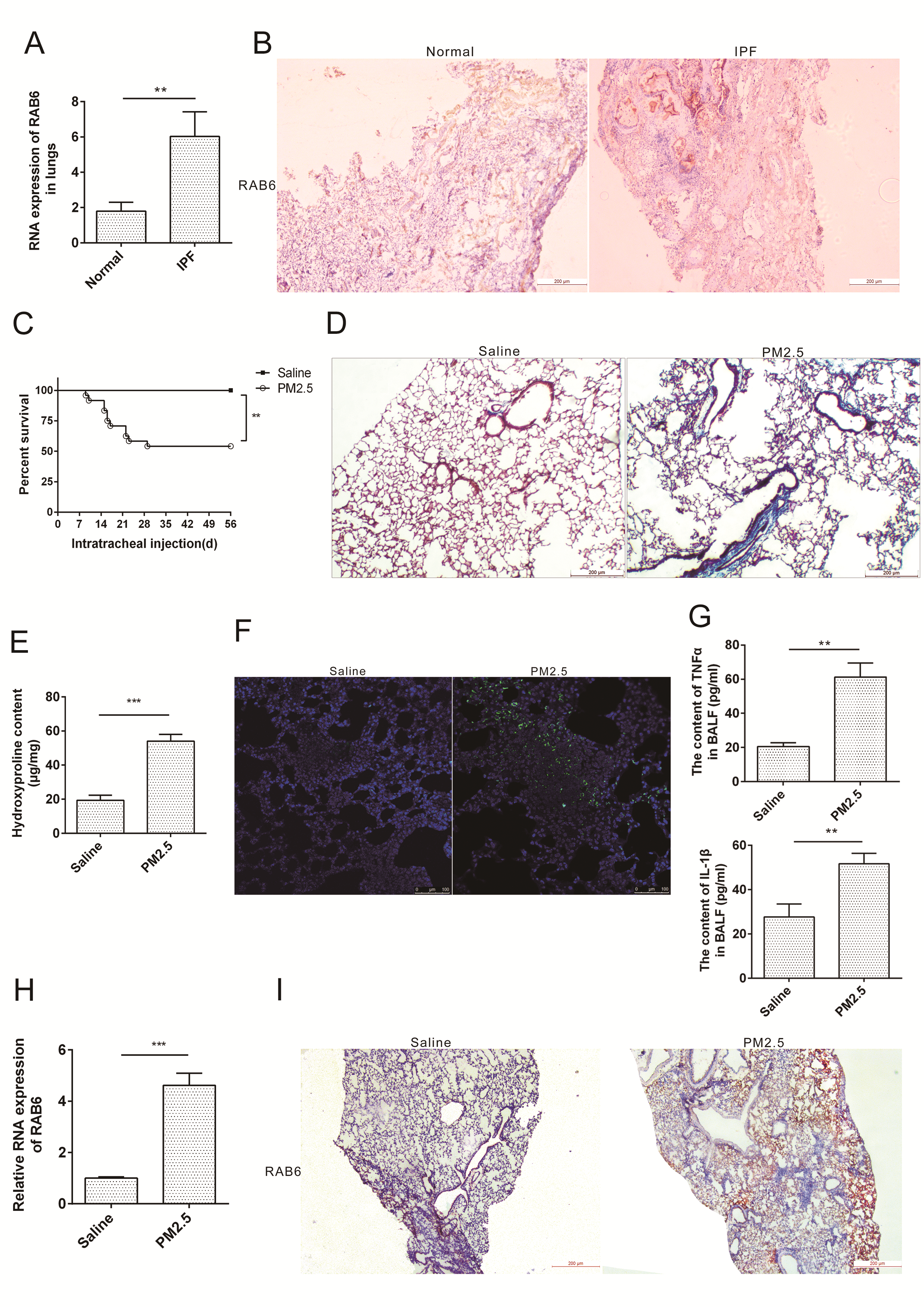

Supplement: Supplementary file 1 — Supplementary Figure 1 [file 41419_2020_3027_MOESM1_ESM.png]

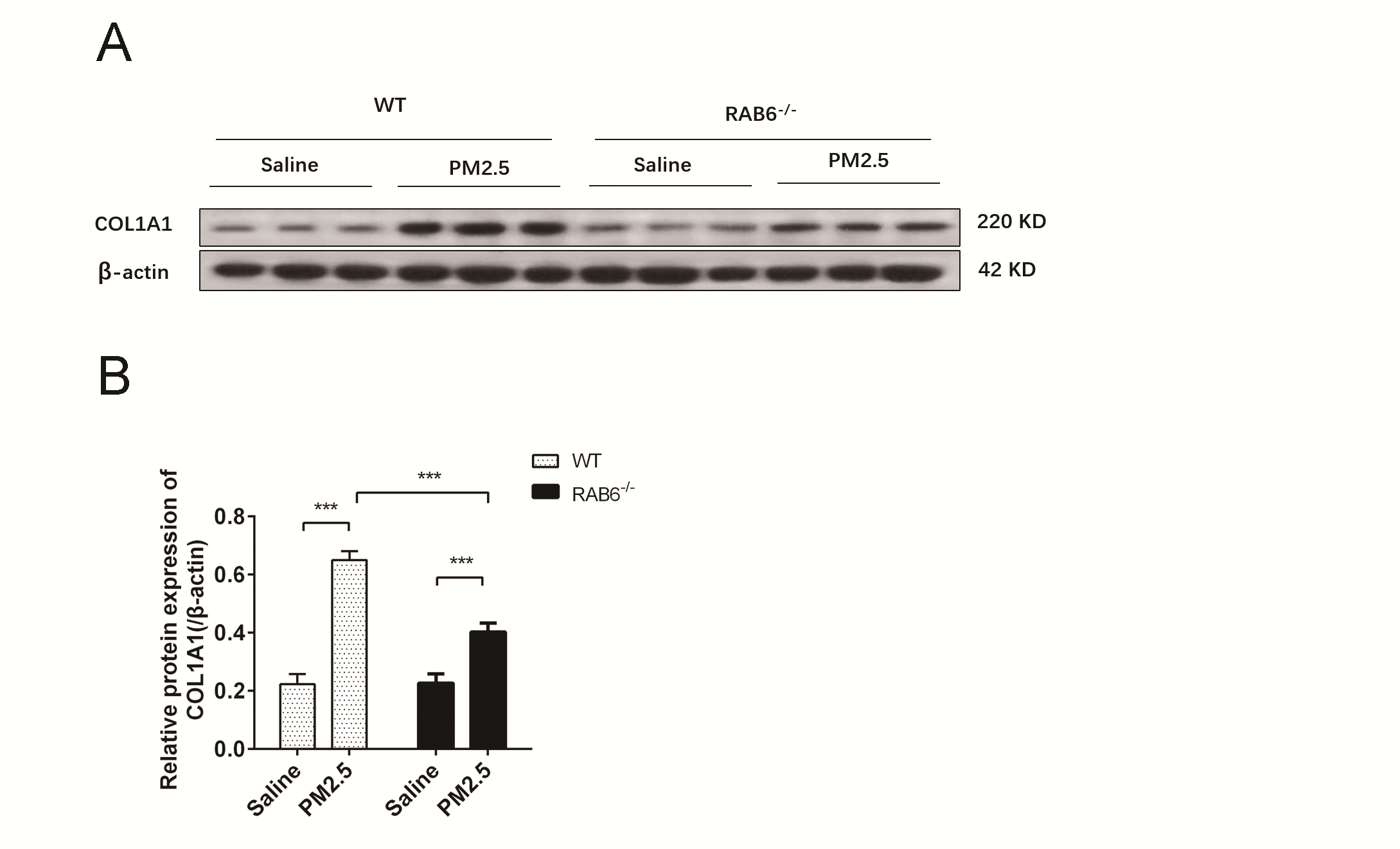

Supplement: Supplementary file 2 — Supplementary Figure 2 [file 41419_2020_3027_MOESM2_ESM.png]

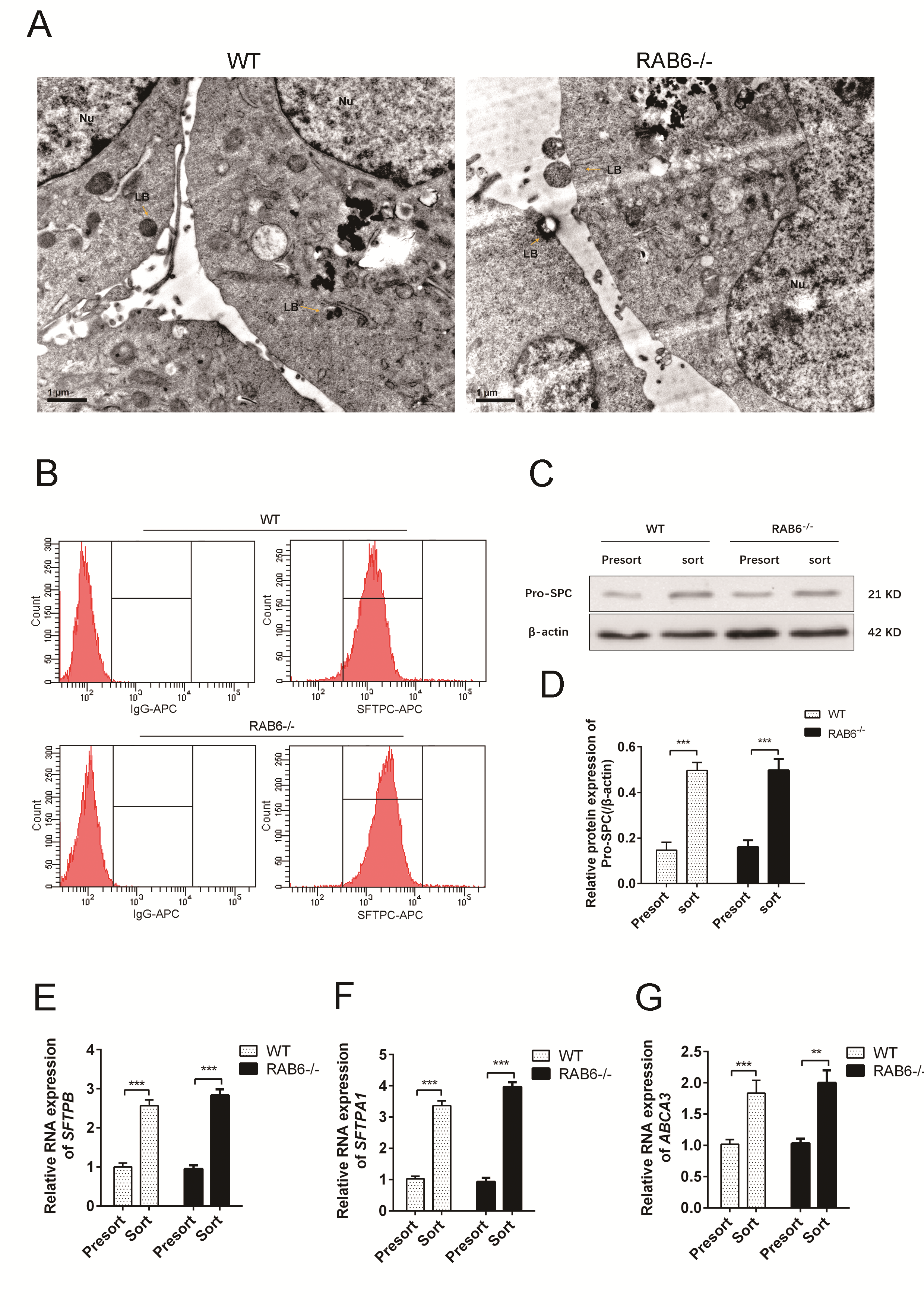

Supplement: Supplementary file 3 — Supplementary Figure 3 [file 41419_2020_3027_MOESM3_ESM.png]

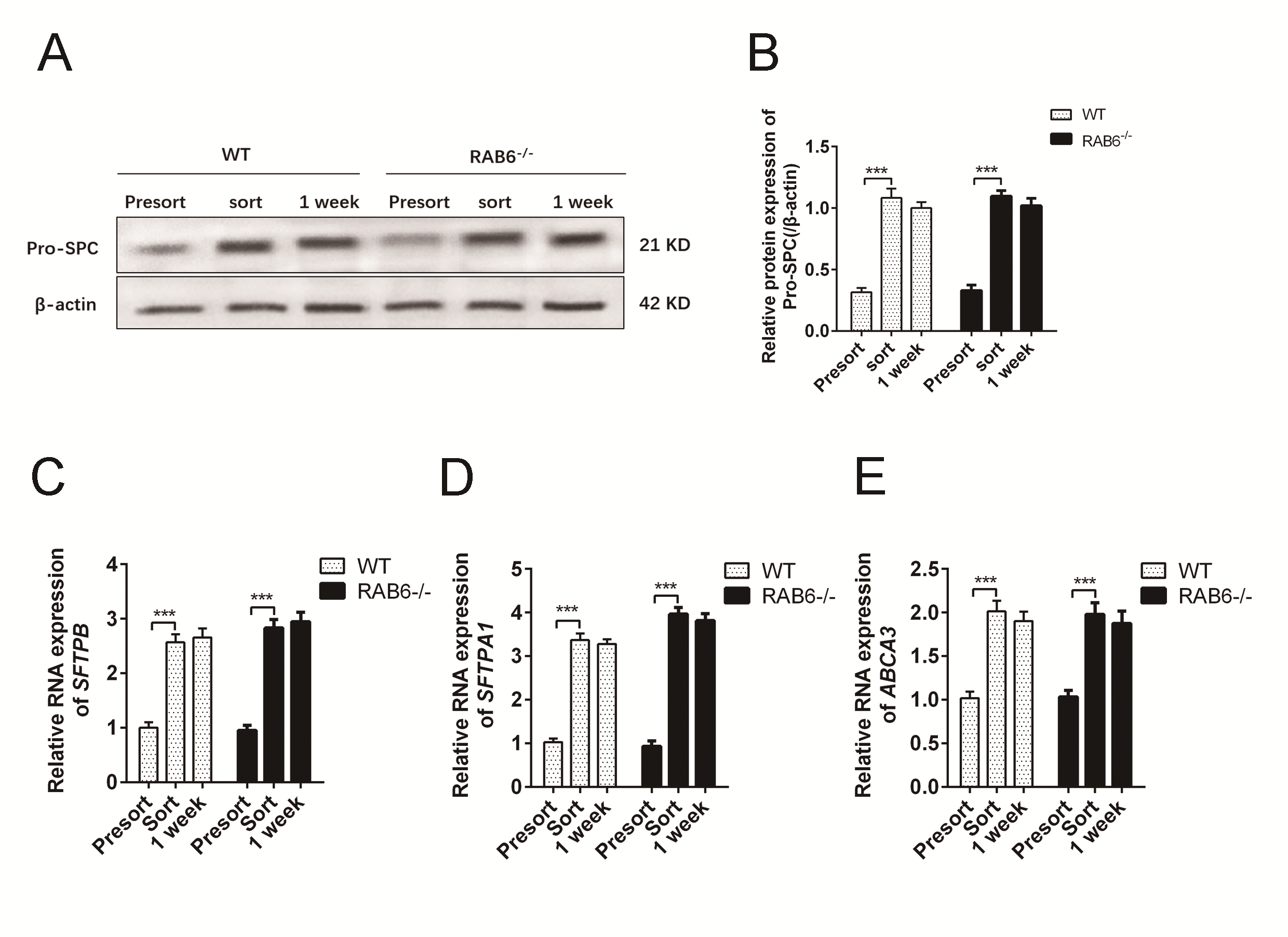

Supplement: Supplementary file 4 — Supplementary Figure 4 [file 41419_2020_3027_MOESM4_ESM.png]

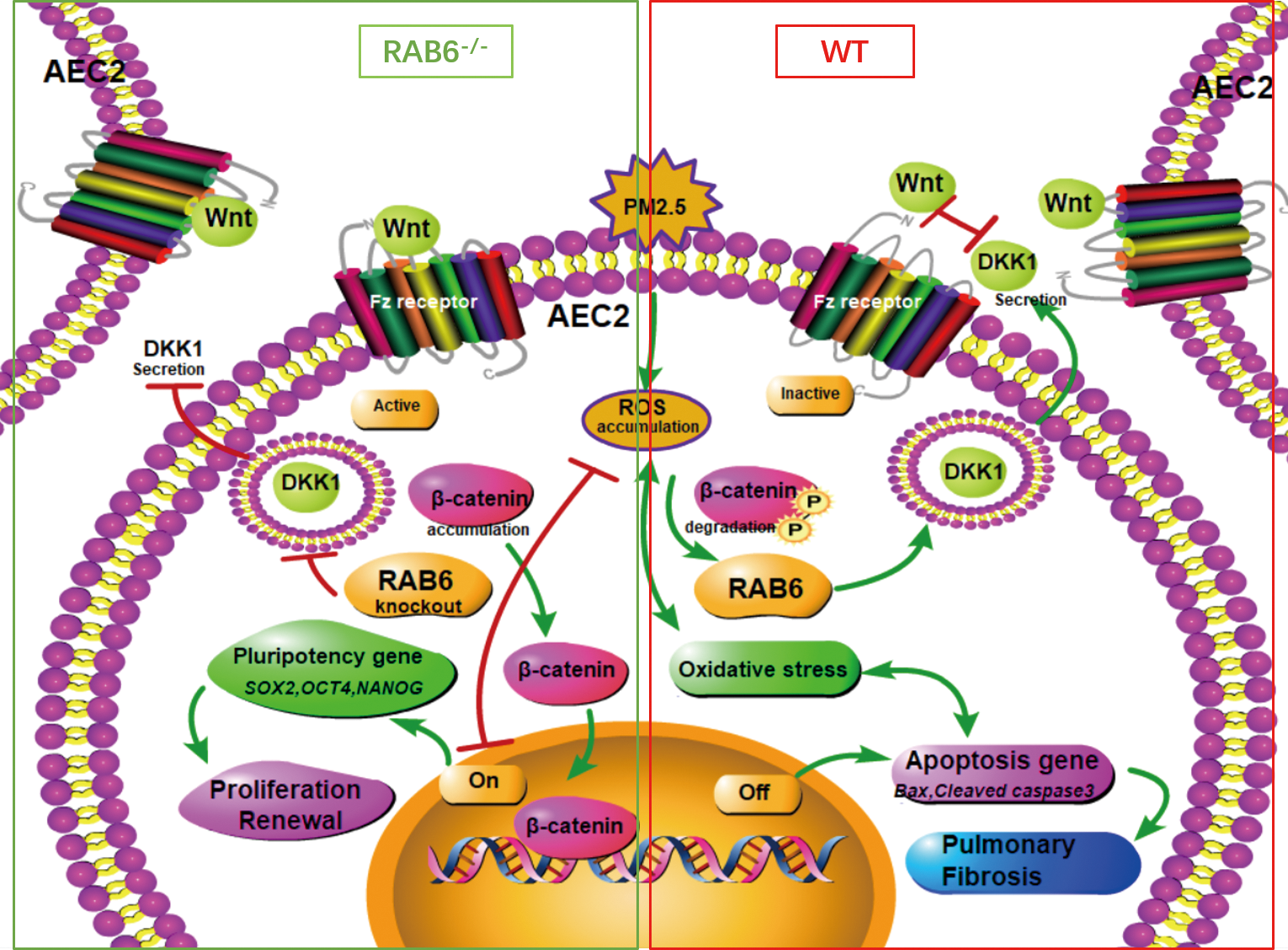

Supplement: Supplementary file 5 — Supplementary Figure 5 [file 41419_2020_3027_MOESM5_ESM.png]
