# Supplementary material for: Small GTPase RAB6 deficiency promotes alveolar progenitor cell renewal and attenuates PM2.5-induced lung injury and fibrosis
Source: Cell Death Dis. 2020 Oct 4;11(10):827. doi: 10.1038/s41419-020-03027-2 (PMC7533251; doi:10.1038/s41419-020-03027-2)
Supplement: Supplementary file 6 — Supplementary Figure Legends [file 41419_2020_3027_MOESM6_ESM.docx]

**Supplementary Figure Legends**

**Supplementary Figure 1. RAB6 was upregulated in IPF and PM2.5-induced lung fibrosis.** (A) The relative expression of RAB6 RNA in IPF tissues and normal lung tissues was measured by qRT-PCR. (B) The expression of RAB6 protein in IPF tissues and normal lung tissues was determined by immunohistochemistry (Representative micrograph of three individual subjects. Scale bar=200 μm). (C) Percentages of surviving PM2.5-injuried mice (n = 24) and Saline treated mice (n = 24) plotted over a 8-week period after intratracheal treatment. ***P* < 0.01 by log-rank test. (D, E) WT mice were exposed to saline or PM2.5(100 mg/kg), collagen content in mouse lung tissue was determined by MASSON staining (D) and hydroxyproline detection (E) (Represented micrographs from 6 mice per group are shown, Scale bar=200 μm). (F) The apoptosis of alveolar epithelial cells in mice was determined by TUNEL staining (Represented micrographs from 6 mice per group are shown, Scale bar=100 μm). (G) The content of IL-1β and TNFα in bronchoalveolar lavage fluid (BALF) were determined by ELISA. (H) The relative expression of RAB6 RNA in lung tissue of mice exposed to PM2.5 or saline was measured by qRT-PCR. (I) The expression of RAB6 protein in lung tissue of mice exposed to PM2.5 or saline was determined by immunohistochemistry (Representative micrograph of six individual subjects. Scale bar=200 μm). (n=6; Unpaired 2-tailed *t* test. ***P* < 0.01; ****P* < 0.001. Bar graphs represent the mean ± SEM for A, E, G and H).

**Supplementary Figure 2. Detection of COL1A1 protein expression in lung tissue.** (A) The expression of COL1A1 protein in the lung tissues of WT and RAB6^-/-^ mice (treated with Saline or PM2.5) was detected by by western blotting. (B) Statistical analysis of relative expression levels of COL1A1 protein. (n=6; Unpaired 2-tailed *t* test. ****P* < 0.001. Bar graphs represent the mean ± SEM).

**Supplementary Figure 3. Identification of AEC2 cells isolated from lung tissues of WT and RAB6^-/-^ mice.** (A) The ultrastructure of AEC2 cells isolated from lung tissues of WT and RAB6^-/-^ mice was detected by transmission electron microscopy (TEM) (Nu, nucleus; LB, lamellar body) (Scale bar = 1 μm). (B) The proportion of SFTPC positive cells isolated from lung tissues of WT and RAB6^-/-^ mice was detected by flow cytometry (Representative FACS images are shown, n=3). (C) The expression of pro-SPC protein in lung cells (presort) and AEC2s (sort) was determined by western blot analysis. (D) Statistical analysis of relative expression levels of pro-SPC protein. (E, F, G) The relative RNA expression of SFTPA1 (E), SFTPB (J) and ABCA3 (G) in lung cells (presort) and AEC2s (sort) was measured by qRT-PCR. (n=3; Unpaired 2-tailed *t* test. ***P* < 0.01; ****P* < 0.001. Bar graphs represent the mean ± SEM for D, E, F and G).

**Supplementary Figure 4. Expression of alveolar transcript and pro-SPC protein after AEC2 cell cultured for 1 week under experimental conditions.** (C) The expression of pro-SPC protein in “presort” AEC2s (unisolated), “sort” AEC2s (isolate and culture for 2 day) and “1 week” AEC2s (culture for 1 week) was determined by western blot analysis. (B) Statistical analysis of relative expression levels of pro-SPC protein. (C, D, E) The relative RNA expression of SFTPA1 (C), SFTPB (D) and ABCA3 (E) in “presort” AEC2s (unisolated), “sort” AEC2s (isolate and culture for 2 day) and “1 week” AEC2s was measured by qRT-PCR. (n=3; Unpaired 2-tailed *t* test. ****P* < 0.001. Bar graphs represent the mean ± SEM for B, C, D and E).

**Supplementary Figure 5. Regulation of RAB6 in AEC2 cells.** The schematic diagram shows the underlying molecular mechanism that RAB6 is involved in PM2.5-induced lung fibrosis by regulating DKK1 secretion and β-catenin signaling, regulating AEC2 cell proliferation and self-renewal.
