# Supplementary material for: Small GTPase RAB6 deficiency promotes alveolar progenitor cell renewal and attenuates PM2.5-induced lung injury and fibrosis
Source: Cell Death Dis. 2020 Oct 4;11(10):827. doi: 10.1038/s41419-020-03027-2 (PMC7533251; doi:10.1038/s41419-020-03027-2)
Supplement: Supplementary file 7 — Supplementary Table 1 [file 41419_2020_3027_MOESM7_ESM.docx]

Table 1. qRT-PCR primer

| Name | Primer sequence（5`-3`） |
| --- | --- |
| *COL1A1* | F:CTGGCGGTTCAGGTCCAAT |
|  | R:TTCCAGGCAATCCACGAGC |
| *ACTA2* | F:CCCAGACATCAGGGAGTAATGG |
|  | R:TCTATCGGATACTTCAGCGTCA |
| SFTPB | F:CACCTCCTCACAAAGATGACC |
|  | R:AAGCAGCTTCAAGGGAAGGAT |
| SFTPC | F:TCCTCGTTGTCGTGGTGATTG |
|  | R:GGAAAAGGTAGCGATGGTGTC |
| SFTPA1 | F:CATCAGATTCTGCAAACAATGGG |
|  | R:GGCTCTGGTACACATCTCTCTAA |
| ABCA3 | F:CGAGGACTACATTCGCTATGAC |
|  | R:GTCCGGGGCTTGGAAAAAGT |
| *DKK1* | F:TGCATGAGGCACGCTATGTG |
|  | R:GCGGCGTTGTGGTCATTAC |
| *SOX2* | F:CGGCACAGATGCAACCGAT |
|  | R: CCGTTCATGTAGGTCTGCG |
| *NANOG* | F:CACAGTTTGCCTAGTTCTGAGG |
|  | R:GCAAGAATAGTTCTCGGGATGAA |
| *OCT4（Pou5f1）* | F:CGGAAGAGAAAGCGAACTAGC |
|  | R:ATTGGCGATGTGAGTGATCTG |
| *GAPDH* | F:TGACCTCAACTACATGGTCTACA |
|  | R:CTTCCCATTCTCGGCCTTG |
