# Supplementary material for: Small GTPase RAB6 deficiency promotes alveolar progenitor cell renewal and attenuates PM2.5-induced lung injury and fibrosis
Source: Cell Death Dis. 2020 Oct 4;11(10):827. doi: 10.1038/s41419-020-03027-2 (PMC7533251; doi:10.1038/s41419-020-03027-2)
Supplement: Supplementary file 8 — Supplementary Table 2 [file 41419_2020_3027_MOESM8_ESM.docx]

**Table 2. Source of antibodies used in the study**

| **Name** | **No** | **Company** |
| --- | --- | --- |
| α-SMA | 19245 | CST |
| DKK1 | 48367 | CST |
| Bax | 5023 | CST |
| Bcl-2 | 3498 | CST |
| Cleaved Caspase-3 | 9664 | CST |
| Wnt3a | 2721 | CST |
| β-catenin | 9582 | CST |
| RAB6 | 9625 | CST |
| c-Myc | 5605 | CST |
| Sox2 | 3579 | CST |
| Histone H3 | 4499 | CST |
| GAPDH | 5174 | CST |
| OGG1 | NB100-106 | NOVUS |
| SFTPC | BA2831-2 | BOSTER |
| β-actin | sc-47778 | Santa |
| PRDX5 | sc-133072 | Santa |
| 8-OHDG | sc-393871 | Santa |
| DKK1 | sc-374574 | Santa |
| SFTPC | sc-518029 | Santa |
| SFTPC-APC | sc-518029 | Santa |
| CD24-PE | 12-0242-82 | eBioscience |
| CD31-Biotin | 13-0311-82 | eBioscience |
| CD34-Biotin | 13-0341-82 | eBioscience |
| CD45-Biotin | 13-0451-82 | eBioscience |
| EpCAM-PE-Cy7 | 25-5791-80 | eBioscience |
| Goat anti-Rabbit IgG FITC | 31635 | Invitrogen |
| Goat anti-Mouse IgG Alexa 647 | A-21235 | Invitrogen |
| Goat anti-Rabbit IgG Secondary Antibody, Biotin | 65-6140 | Invitrogen |
