# Supplementary material for: Small GTPase RAB6 deficiency promotes alveolar progenitor cell renewal and attenuates PM2.5-induced lung injury and fibrosis
Source: Cell Death Dis. 2020 Oct 4;11(10):827. doi: 10.1038/s41419-020-03027-2 (PMC7533251; doi:10.1038/s41419-020-03027-2)
Supplement: Supplementary file 9 — Supplementary Table 3 [file 41419_2020_3027_MOESM9_ESM.docx]

Table 3.1 Description of statistical analysis results (Figure1-8)

| Figure | Compared group | Significant? | Summary | P Value |
| --- | --- | --- | --- | --- |
| Figure 1-B | Comparison of Survival Curves |  | * | 0.032 |
| Figure 1-D | Saline-WT vs. PM2.5-WT | Yes | *** | < 0.0001 |
|  | Saline-RAB6^-/-^ vs. PM2.5-RAB6^-/-^ | Yes | * | 0.0296 |
|  | PM2.5-WT vs. PM2.5-RAB6^-/-^ | Yes | *** | 0.0003 |
| Figure 1-E | Saline-WT vs. PM2.5-WT | Yes | *** | < 0.0001 |
|  | Saline-RAB6^-/-^ vs. PM2.5-RAB6^-/-^ | Yes | *** | < 0.0001 |
|  | PM2.5-WT vs. PM2.5-RAB6^-/-^ | Yes | *** | < 0.0001 |
| Figure 1-F | Saline-WT vs. PM2.5-WT | Yes | *** | < 0.0001 |
|  | Saline-RAB6^-/-^ vs. PM2.5-RAB6^-/-^ | Yes | *** | < 0.0001 |
|  | PM2.5-WT vs. PM2.5-RAB6^-/-^ | Yes | *** | < 0.0001 |
| Figure 1-G | Saline-WT vs. PM2.5-WT | Yes | *** | < 0.0001 |
|  | Saline-RAB6^-/-^ vs. PM2.5-RAB6^-/-^ | Yes | *** | 0.0003 |
|  | PM2.5-WT vs. PM2.5-RAB6^-/-^ | Yes | *** | < 0.0001 |
| Figure 1-H | Saline-WT vs. PM2.5-WT | Yes | *** | < 0.0001 |
|  | Saline-RAB6^-/-^ vs. PM2.5-RAB6^-/-^ | Yes | *** | < 0.0001 |
|  | PM2.5-WT vs. PM2.5-RAB6^-/-^ | Yes | *** | < 0.0001 |
| Figure 1-I | Saline-WT vs. PM2.5-WT | Yes | *** | < 0.0001 |
|  | Saline-RAB6^-/-^ vs. PM2.5-RAB6^-/-^ | Yes | *** | < 0.0001 |
|  | PM2.5-WT vs. PM2.5-RAB6^-/-^ | Yes | *** | < 0.0001 |
| Figure 1-K | Saline-WT vs. PM2.5-WT | Yes | *** | < 0.0001 |
|  | Saline-RAB6^-/-^ vs. PM2.5-RAB6^-/-^ | Yes | *** | < 0.0001 |
|  | PM2.5-WT vs. PM2.5-RAB6^-/-^ | Yes | *** | < 0.0001 |
| Figure 2-B | Saline-WT vs. PM2.5-WT | Yes | *** | < 0.0001 |
|  | Saline-RAB6^-/-^ vs. PM2.5-RAB6^-/-^ | Yes | *** | < 0.0001 |
|  | PM2.5-WT vs. PM2.5-RAB6^-/-^ | Yes | *** | < 0.0001 |
| Figure 2-C | Saline-WT vs. PM2.5-WT | Yes | *** | < 0.0001 |
|  | Saline-RAB6^-/-^ ***vs. PM2.5-RAB6-/-*** | Yes | *** | < 0.0001 |
|  | PM2.5-WT vs. PM2.5-RAB6^-/-^ | Yes | *** | < 0.0001 |
| Figure 2-D | Saline-WT vs. PM2.5-WT | Yes | *** | < 0.0001 |
|  | Saline-RAB6^-/-^ vs. PM2.5-RAB6^-/-^ | Yes | *** | < 0.0001 |
|  | PM2.5-WT vs. PM2.5-RAB6^-/-^ | Yes | *** | < 0.0001 |
| Figure 2-E | Saline-WT vs. PM2.5-WT | Yes | *** | < 0.0001 |
|  | Saline-RAB6^-/-^ ***vs. PM2.5-RAB6-/-*** | Yes | *** | < 0.0001 |
|  | PM2.5-WT vs. PM2.5-RAB6^-/-^ | Yes | *** | < 0.0001 |
| Figure 2-G |  |  |  |  |
| 8-OHDG | Saline-WT vs. PM2.5-WT | Yes | *** | < 0.0001 |
|  | Saline-RAB6^-/-^ ***vs. PM2.5-RAB6-/-*** | Yes | *** | < 0.0001 |
|  | PM2.5-WT vs. PM2.5-RAB6^-/-^ | Yes | *** | < 0.0001 |
| PRDX5 | Saline-WT vs. PM2.5-WT | Yes | *** | < 0.0001 |
|  | Saline-RAB6^-/-^ ***vs. PM2.5-RAB6-/-*** | Yes | *** | < 0.0001 |
|  | PM2.5-WT vs. PM2.5-RAB6^-/-^ | Yes | *** | < 0.0001 |
| OGG1 | Saline-WT vs. PM2.5-WT | Yes | *** | < 0.0001 |
|  | Saline-RAB6^-/-^ ***vs. PM2.5-RAB6-/-*** | Yes | *** | < 0.0001 |
|  | PM2.5-WT vs. PM2.5-RAB6^-/-^ | Yes | *** | < 0.0001 |
| Figure 3-B | Saline-WT vs. PM2.5-WT | Yes | *** | < 0.0001 |
|  | Saline-RAB6^-/-^ vs. PM2.5-RAB6^-/-^ | Yes | *** | < 0.0001 |
|  | PM2.5-WT vs. PM2.5-RAB6^-/-^ | Yes | *** | 0.0002 |
|  |  |  |  |  |
| Figure 3-D | Saline-WT vs. PM2.5-WT | Yes | *** | < 0.0001 |
|  | Saline-RAB6^-/-^ ***vs. PM2.5-RAB6-/-*** | Yes | *** | < 0.0001 |
|  | PM2.5-WT vs. PM2.5-RAB6^-/-^ | Yes | *** | < 0.0001 |
| Figure 3-G |  |  |  |  |
| RAB6 | Saline-WT vs. PM2.5-WT | Yes | *** | < 0.0001 |
|  | PM2.5-WT vs. PM2.5-RAB6^-/-^ | Yes | *** | < 0.0001 |
| Bax | Saline-WT vs. PM2.5-WT | Yes | *** | < 0.0001 |
|  | Saline-RAB6^-/-^ vs. PM2.5-RAB6^-/-^ | Yes | *** | < 0.0001 |
|  | PM2.5-WT vs. PM2.5-RAB6^-/-^ | Yes | *** | < 0.0001 |
| Bcl2 | Saline-WT vs. PM2.5-WT | Yes | *** | < 0.0001 |
|  | Saline-RAB6^-/-^ vs. PM2.5-RAB6^-/-^ | Yes | *** | < 0.0001 |
|  | PM2.5-WT vs. PM2.5-RAB6^-/-^ | Yes | *** | < 0.0001 |
| caspase3 | Saline-WT vs. PM2.5-WT | Yes | *** | < 0.0001 |
|  | Saline-RAB6^-/-^ vs. PM2.5-RAB6^-/-^ | Yes | *** | < 0.0001 |
|  | PM2.5-WT vs. PM2.5-RAB6^-/-^ | Yes | *** | < 0.0001 |
| Figure 4-D | Saline-WT vs. PM2.5-WT | Yes | *** | < 0.0001 |
|  | Saline-RAB6^-/-^ vs. PM2.5-RAB6^-/-^ | Yes | *** | < 0.0001 |
|  | PM2.5-WT vs. PM2.5-RAB6^-/-^ | Yes | *** | < 0.0001 |
|  |  |  |  |  |
| Figure 4-F | Saline-WT vs. PM2.5-WT | Yes | ** | 0.0039 |
|  | Saline-RAB6^-/-^ vs. PM2.5-RAB6-/- | Yes | *** | 0.0006 |
|  | PM2.5-WT vs. PM2.5-RAB6^-/-^ | Yes | ** | 0.004 |
|  |  |  |  |  |
| Figure 4-H | Saline-WT vs. PM2.5-WT | Yes | ** | 0.0011 |
|  | Saline-RAB6^-/-^ vs. PM2.5-RAB6^-/-^ | Yes | *** | 0.0002 |
|  | PM2.5-WT vs. PM2.5-RAB6^-/-^ | Yes | *** | < 0.0001 |
| Figure 4-I | Saline-WT vs. PM2.5-WT | Yes | ** | 0.0036 |
|  | Saline-RAB6^-/-^ vs. PM2.5-RAB6^-/-^ | Yes | ** | 0.0018 |
|  | PM2.5-WT vs. PM2.5-RAB6^-/-^ | Yes | *** | 0.0004 |
| Figure 4-J | Saline-WT vs. PM2.5-WT | Yes | ** | 0.0011 |
|  | Saline-RAB6^-/-^ vs. PM2.5-RAB6^-/-^ | Yes | *** | 0.0007 |
|  | PM2.5-WT vs. PM2.5-RAB6^-/-^ | Yes | *** | 0.0005 |
| Figure 5 B |  |  |  |  |
| wnt3a | Saline-WT vs. PM2.5-WT | Yes | *** | < 0.0001 |
|  | Saline-RAB6^-/-^ vs. PM2.5-RAB6^-/-^ | Yes | *** | < 0.0001 |
|  |  |  |  |  |
| B-catenin | Saline-WT vs. PM2.5-WT | Yes | *** | < 0.0001 |
|  | Saline-RAB6^-/-^ vs. PM2.5-RAB6^-/-^ | Yes | *** | < 0.0001 |
|  | PM2.5-WT vs. PM2.5-RAB6^-/-^ |  | ** | 0.0039 |
|  |  |  |  |  |
| DKK1 | Saline-WT vs. PM2.5-WT | Yes | *** | < 0.0001 |
|  | PM2.5-WT vs. PM2.5-RAB6^-/-^ | Yes | *** | < 0.0001 |
|  |  |  |  |  |
| C-MYC | Saline-WT vs. PM2.5-WT | Yes | *** | < 0.0001 |
|  | Saline-RAB6^-/-^ vs. PM2.5-RAB6^-/-^ | Yes | *** | < 0.0001 |
|  | PM2.5-WT vs. PM2.5-RAB6^-/-^ |  | ** | 0.0093 |
| RAB6 | Saline-WT vs. PM2.5-WT | Yes | *** | < 0.0001 |
|  | PM2.5-WT vs. PM2.5-RAB6^-/-^ | Yes | *** | < 0.0001 |
|  |  |  |  |  |
| Figure 5-G | Saline-WT vs. Saline-RAB6^-/-^ | Yes | *** | < 0.0001 |
|  | PM2.5-WT vs. PM2.5-RAB6^-/-^ | Yes | *** | < 0.0001 |
| Figure 6-B | PBS-WT vs. PBS-RAB6^-/-^ | Yes | *** | < 0.0001 |
|  | PBS-RAB6^-/-^ vs. DKK1 protein-RAB6^-/-^ | Yes | *** | < 0.0001 |
|  |  |  |  |  |
| Figure 6-D | PBS-WT vs. PBS-RAB6^-/-^ | Yes | *** | 0.0001 |
|  |  |  | * | 0.0135 |
|  | PBS-RAB6^-/-^ vs. DKK1 protein-RAB6^-/-^ | Yes | *** | 0.0003 |
| Figure 6-F |  |  |  |  |
| B-catenin | PBS-WT vs. PBS-RAB6^-/-^ | Yes | *** | 0.0153 |
|  | PBS-RAB6^-/-^ vs. DKK1 protein-RAB6^-/-^ | Yes | *** | 0.0012 |
|  |  |  |  |  |
| C-MYC | PBS-WT vs. PBS-RAB6^-/-^ | Yes | *** | 0.0002 |
|  | PBS-RAB6^-/-^ vs. DKK1 protein-RAB6^-/-^ | Yes | *** | < 0.0001 |
|  |  |  |  |  |
| Figure 6-H | PBS-WT vs. PBS-RAB6^-/-^ | Yes | ** | 0.0015 |
|  | PBS-RAB6^-/-^ vs. DKK1 protein-RAB6^-/-^ | Yes | *** | < 0.0001 |
|  |  |  |  |  |
| Figure 6-I | PBS-WT vs. PBS-RAB6^-/-^ | Yes | *** | < 0.0001 |
|  | PBS-RAB6^-/-^ vs. DKK1 protein-RAB6^-/-^ | Yes | *** | < 0.0001 |
| Figure 6-J | PBS-WT vs. PBS-RAB6^-/-^ | Yes | *** | < 0.0001 |
|  | PBS-RAB6^-/-^ vs. DKK1 protein-RAB6^-/-^ | Yes | *** | < 0.0001 |
| Figure 6-K | PBS-WT vs. PBS-RAB6^-/-^ | Yes | *** | < 0.0001 |
|  | PBS-RAB6^-/-^ vs. DKK1 protein-RAB6^-/-^ | Yes | *** | < 0.0001 |
| Figure 7-B |  |  |  |  |
| RAB6 | PBS-NC vs. PBS-RAB6 | Yes | *** | < 0.0001 |
|  | Gallocyanine-NC vs. Gallocyanine-RAB6 | Yes | *** | < 0.0001 |
| DKK1 | PBS-NC vs. PBS-RAB6 | Yes | ** | 0.0028 |
|  | Gallocyanine-NC vs. Gallocyanine-RAB6 | Yes | *** | 0.0007 |
| B-catenin | PBS-NC vs. PBS-RAB6 | Yes | *** | < 0.0001 |
|  | PBS-RAB6 vs. Gallocyanine-RAB6 | Yes | *** | < 0.0001 |
| SOX2 | PBS-NC vs. PBS-RAB6 | Yes | *** | < 0.0001 |
|  | PBS-RAB6 vs. Gallocyanine-RAB6 | Yes | *** | < 0.0001 |
|  |  |  |  |  |
| Figure 7-D | PBS-NC vs. PBS-RAB6 | Yes | *** | < 0.0001 |
|  | Gallocyanine-NC vs. Gallocyanine-RAB6 | Yes | *** | < 0.0001 |
|  |  |  |  |  |
| Figure 7-F | PBS-NC vs. PBS-RAB6 | Yes | *** | 0.0007 |
|  | PBS-RAB6 vs. Gallocyanine-RAB6 | Yes | *** | 0.0001 |
|  |  |  |  |  |
| Figure 7-G | PBS-NC vs. PBS-RAB6 | Yes | ** | 0.0016 |
|  | PBS-RAB6 vs. Gallocyanine-RAB6 | Yes | *** | 0.0002 |
|  |  |  |  |  |
| Figure 7-I | PBS-NC vs. PBS-RAB6 | Yes | *** | 0.0006 |
|  | PBS-RAB6 vs. Gallocyanine-RAB6 | Yes | ** | 0.0018 |
|  |  |  |  |  |
| Figure 7-J | PBS-NC vs. PBS-RAB6 | Yes | *** | 0.0001 |
|  | PBS-RAB6 vs. Gallocyanine-RAB6 | Yes | *** | < 0.0001 |
| Figure 7-L | PBS-NC vs. PBS-RAB6 | Yes | *** | < 0.0001 |
|  | PBS-RAB6 vs. Gallocyanine-RAB6 | Yes | *** | < 0.0001 |
| Figure 7-K | PBS-NC vs. PBS-RAB6 | Yes | *** | < 0.0001 |
|  | PBS-RAB6 vs. Gallocyanine-RAB6 | Yes | *** | < 0.0001 |
| Figure 8-B | PM2.5 vs. PM2.5+Gallocyanine | Yes | *** | 0.0003 |
|  |  |  |  |  |
| Figure 8-C | PM2.5 vs. PM2.5+Gallocyanine | Yes | *** | < 0.0001 |
|  |  |  |  |  |
| Figure 8-D | PM2.5 vs. PM2.5+Gallocyanine | Yes | *** | < 0.0001 |
|  |  |  |  |  |
| Figure 8-F | PM2.5 vs. PM2.5+Gallocyanine | Yes | *** | < 0.0001 |
|  |  |  |  |  |
| Figure 8-G | PM2.5 vs. PM2.5+Gallocyanine | Yes | *** | < 0.0001 |

All P values less than 0.001 are summarized with three asterisks, with no possibility of four asterisks.

Table 3.2 Description of statistical analysis results (Figure S1-S4)

| Supplementary Figure | Compared group | Significant? | Summary | P Value |
| --- | --- | --- | --- | --- |
| Figure-S1 |  |  |  |  |
| A | Normal vs IPF | Yes | ** | 0.0025 |
|  |  |  |  |  |
| E | Saline vs PM2.5 | Yes | *** | 0.0003 |
|  |  |  |  |  |
| G | Saline vs PM2.5 | Yes | ** | 0.0053 |
|  | Saline vs PM2.5 | Yes | ** | 0.0011 |
| H | Saline vs PM2.5 | Yes | *** | 0.0002 |
| Figure-S2 |  |  |  |  |
|  | Saline-WT vs. PM2.5-WT | Yes | *** | < 0.0001 |
|  | Saline-RAB6^-/-^ vs. PM2.5-RAB6^-/-^ | Yes | *** | < 0.0001 |
| Figure-S3 |  |  |  |  |
| D | Presort-WT vs. sort-WT | Yes | *** | 0.0003 |
|  | Presort-RAB6^-/-^ vs. sort-RAB6^-/-^ | Yes | *** | 0.0006 |
|  |  |  |  |  |
| E | Presort-WT vs. sort-WT | Yes | *** | 0.0001 |
|  | Presort-RAB6^-/-^ vs. sort-RAB6^-/-^ | Yes | *** | < 0.0001 |
|  |  |  |  |  |
| F | Presort-WT vs. sort-WT | Yes | *** | < 0.0001 |
|  | Presort-RAB6^-/-^ vs. sort-RAB6^-/-^ | Yes | *** | < 0.0001 |
|  |  |  |  |  |
| G | Presort-WT vs. sort-WT | Yes | *** | 0.0004 |
|  | Presort-RAB6^-/-^ vs. sort-RAB6^-/-^ | Yes | ** | 0.0014 |
| Figure-S4 |  |  |  |  |
| B | Presort-WT vs. sort-WT | Yes | *** | < 0.0001 |
|  | Presort-RAB6^-/-^ vs. sort-RAB6^-/-^ | Yes | *** | < 0.0001 |
|  |  |  |  |  |
| C | Presort-WT vs. sort-WT | Yes | *** | 0.0001 |
|  | Presort-RAB6^-/-^ vs. sort-RAB6^-/-^ | Yes | *** | < 0.0001 |
|  |  |  |  |  |
| D | Presort-WT vs. sort-WT | Yes | *** | < 0.0001 |
|  | Presort-RAB6^-/-^ vs. sort-RAB6^-/-^ | Yes | *** | < 0.0001 |
|  |  |  |  |  |
| E | Presort-WT vs. sort-WT | Yes | *** | 0.0003 |
|  | Presort-RAB6^-/-^ vs. sort-RAB6^-/-^ | Yes | *** | 0.0004 |

All P values less than 0.001 are summarized with three asterisks, with no possibility of four asterisks.
